# Do more birds mean more bird-aircraft collisions? A meta-analysis testing a key wildlife management tenet

**DOI:** 10.1371/journal.pone.0349352

**Published:** 2026-07-01

**Authors:** Shane Guenin, Yue Liu, Bradley F. Blackwell, Travis L. DeVault, Esteban Fernández-Juricic

**Affiliations:** 1 Department of Biological Sciences, Purdue University, West Lafayette, Indiana, United States of America; 2 APHIS - Wildlife Services, U.S. Department of Agriculture, Sandusky, Ohio, United States of America; 3 Savannah River Ecology Laboratory, University of Georgia, Aiken, South Carolina, United States of America; Universidad Miguel Hernandez de Elche, SPAIN

## Abstract

Bird-aircraft collisions (bird strikes) pose threats to aviation safety and avian life. One of the tenets of airport-based management strategies to prevent bird strikes is the reduction of bird density within established separation distances from air operations areas. The rationale is that higher local bird densities could increase the spatial and temporal overlap in the use of airspace by both birds and aircraft, leading to a higher frequency of bird strikes. However, the strength and direction of this relationship have not been evaluated across published studies. This is an important gap, given how entrenched this assumed relationship has become to allocate limited resources to airport wildlife management. In this study, we assessed the strength of the relationship between avian abundance and bird strikes across studies using a meta-analytic approach. Through a reproducible literature search and screening criteria, we identified 20 outcomes (i.e., effect sizes) from 13 studies. We conducted a multilevel meta-analysis and found a positive correlation (Pearson’s *r* = 0.520, 95% confidence intervals: 0.308–0.683), supporting the positive relationship between bird abundance and bird strike frequency. We additionally found evidence that the existing literature has high levels of between-study heterogeneity and publication bias, low statistical power, and multiple methodological concerns. These issues suggest that our effect size estimation should be interpreted with care. Given the limitations of the published literature testing this relationship, we provide a set of methodological recommendations for improving future experiments. We call for prioritizing the empirical testing of the abundance-bird strike relationship on and near airports across the world, and the standardization of bird survey approaches. These future tests are key to aligning management efforts to local airport needs.

## Introduction

Animal-vehicle collisions represent an evolutionarily novel threat to wildlife populations [1–4] and pose substantial financial and safety hazards [5–7]. For instance, animal-aircraft collisions were estimated to cost over $142 million annually in direct and indirect costs to the civil aviation industry operating within the USA (1990–2017), but actual losses might be double this amount [8; see also 9]. The number of strikes reported annually to the U.S. Federal Aviation Administration (FAA) has increased more than tenfold from 1,850 strikes in 1990 to 22,372 in 2024. Over this period, 319,047 strikes were reported, and birds were involved in more than 90% of those incidents [10].

Bird-aircraft collisions (hereafter, bird strikes) pose threats not only to birds but also to aircraft operations and passenger safety. Perhaps the most well-known bird strike incident in recent history is the 2009 bird strike of an Airbus 320 with 155 passengers on board, which was forced to land in the Hudson River after colliding with a flock of Canada geese (*Branta canadensis*) [11]. From 1988 to 2024, collisions with birds destroyed 360 aircraft and resulted in 643 human deaths worldwide [10]. The Avisure database, with records going back to 1912, attributes the destruction of 681 aircraft and 979 human deaths to bird strikes [12]. The chances of a bird surviving a collision with an aircraft are very slim [13], which raises concerns for threatened or endangered bird species [14,15]. Approximately 71% of all bird strikes reported to the FAA occurred at low altitudes (i.e., ≤ 152 m above ground level), thus likely within the airport environment [8]. Therefore, airports have become focal points of wildlife management to enhance the safety of passengers and, indirectly, minimize avian mortality.

To avoid bird strikes at airports, wildlife managers have been implementing multiple techniques for decades with a specific goal: to reduce the local density of birds at airfields [16–18]. The rationale behind this goal has been rooted in the hypothesis that higher bird population abundances lead to increased local densities, which in an airport environment could increase the frequency of spatial and temporal overlap in the use of airspace by birds and aircraft. This overlap would lead to a higher rate of close encounters and greater risk of bird strikes (see [19]). Therefore, the prediction often relied upon for airport wildlife management is that a higher abundance of birds in an airport environment will be associated with a higher frequency of bird strikes. The implication of this prediction is that controlling bird abundance at airports should reduce the risk of bird strikes [20,21].

This abundance-strike prediction has become widely accepted by airport managers [22]. For example, airports spend considerable human and financial resources to control bird abundance locally [6,23–25]. The abundance-strike prediction has become so widespread that Dolbeer et al. [19] provided a cautionary note to airport wildlife managers about increasing populations of urban-adapted large bird species commonly involved in strikes in the USA (see also [26]). Although a few studies have tested the relationship between bird abundance and bird strike frequency [27–30], evidence from other collision contexts (bird-building collisions) suggests some uncertainty about the role of species abundance in collision-related mortality [31,32].

Consequently, it is essential to synthesize the available information and assess whether the abundance-strike prediction is supported across the available primary literature, and in which contexts. This assessment is relevant for the management of wildlife on and near airports, because the metric of success for many current management strategies is measured in part by the numerical reduction in local bird abundance [33]. If, in the context of airport wildlife management, this prediction does not have widespread support, then other approaches (e.g., modifying behavior of target species, [7,34]) should take a higher management priority to minimize bird strikes. Further, certain ecological or methodological factors can alter the ability to observe such a relationship or confound the abundance-strike relationship, should it exist. For example, flocking birds are more likely to collide with aircraft [35,36], which could affect the risk of species that tend to flock relative to those that do not. If any ecological or design factor affects the effect size, changes in data collection methods should be considered to provide airport biologists the best chance of understanding the situation at their airports.

Meta-analysis is a set of statistical techniques used for synthesizing the results of multiple studies [37,38]. Meta-analyses enable researchers and policy makers to understand the average effects of studies and their variability, which can help make more informed decisions about policy issues [37,38]. Moreover, using a meta-analysis to analyze a system produces unitless measures describing the magnitude of an effect, calculated using raw data that is likely to be in different units, as is the case with bird abundance data in the primary literature. We used a meta-analytic approach to investigate two questions related to the abundance-strike prediction. First, what is the degree of empirical support for and against the abundance-strike prediction based on quantitative tests reported in the literature? Second, what other parameters could affect the association between avian abundance and strike frequency? We assessed factors of both ecological and methodological relevance.

## Methods

### Literature screening and inclusion criteria

We conducted our systematic review and meta-analysis following the guidelines regarding best practices for systematic reviews and meta-analyses in wildlife biology [39], and PRISMA standards (a 27-item checklist) for meta-analyses in ecology and evolution [40]. PRISMA standards provide guidance on finding and extracting data from relevant studies, study selection, analysis methods, and reporting methodology to ensure reproducibility. To include the highest proportion of relevant literature in our meta-analysis, we searched six databases for both peer-reviewed studies and gray literature (unpublished theses and dissertations, agency reports, etc.). Using keyword searches, we searched Web of Science (all databases, https://www.webofscience.com), Google Scholar (https://scholar.google.com), Scopus (https://www.scopus.com), Semantic Scholar (https://www.semanticscholar.org), ProQuest (https://www.proquest.com), and Open Access Theses and Dissertations (https://oatd.org). We also screened reference lists of returned studies for additional relevant papers assessing the association between bird abundance (sometimes reported as “bird density”) and bird strike frequency (sometimes reported as “strike rate”).

The search string used for the Web of Science search (and subsequently modified for other databases) was: “Bird* AND Abundance AND [Airport OR aviation] AND [“bird-aircraft collision*” OR “bird strike*”] AND Regression” and “Bird* AND Density AND [Airport OR aviation] AND [“bird-aircraft collision*” OR “bird strike*”] AND Regression.” If a modified search string yielded invalid results (more than 1,000,000 results or the titles and keywords of the first 50 papers were not relevant to the topic), we continued revising it. There was no year filter applied during our literature search (i.e., our search returned literature published in or before October of 2024). For a full description of the search strings used and the number of articles identified in each database, see S1 Appendix.

**Fig 1:**
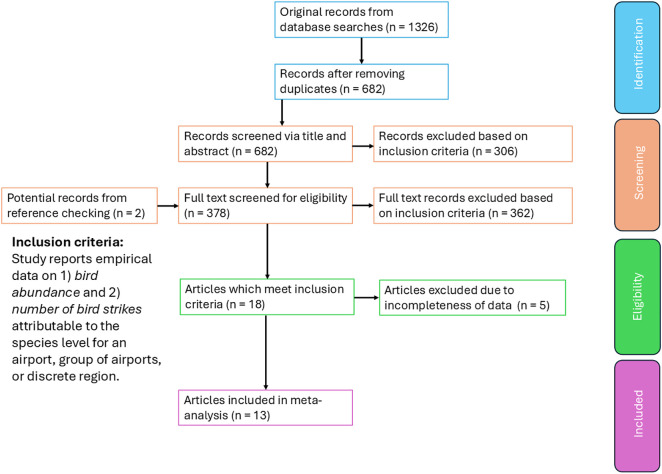
Steps taken to identify, screen, select, and include articles that provided data to test the association between bird abundance and bird strike frequency.

After searching these databases, we obtained a total of 1,225 search results, including publications and unpublished theses (last access: Dec 28th, 2021). We removed 520 duplicates, leaving us with 615 unique records (Fig 1). Coauthor YL performed the initial literature search and screening. A secondary search using the same databases and similar search strings was conducted between Aug 9^th^ – October 28^th^, 2024, to include studies published after 2021 (S1 Appendix) to update the meta-analysis before submission. This last search returned 67 unique records. Coauthor SG screened these articles for inclusion. We included a given article in our meta-analysis if it: (a) directly tested the association between bird abundance and bird strike frequency and reported results, (b) did not directly report all raw data but contained enough information to extract or compute effect size and sample size, or alternatively (c) did not explicitly test a strike-abundance relationship, but provided bird abundance and bird strike data (both expressed as continuous factors) across time or species, allowing us to test the association and estimate an effect size. Studies were excluded if computer-simulated bird abundance data were reported rather than empirical data (e.g., [41]), or if after requesting the original data from the authors, we did not receive a response. We used only English-language studies. We considered different proxies of bird abundance: number of birds, number of nests, number of individuals/km, and percentage occurrence of a species. We considered different proxies of bird strike frequency: the number of bird strikes, bird strike rate (number of strikes per unit time or aircraft movement), and the percentage of a particular species in total bird strike incidents. Using multiple proxies of bird abundance and bird strike frequency allowed us to maximize the sample size in our meta-analysis. Throughout the rest of the manuscript, we refer to these proxies as bird abundance and bird strike frequency for simplicity. Fig 1 shows the process we followed to refine our dataset using the eligibility criteria described above.

After the screening process, we identified 13 studies which met our inclusion criteria [27–30,42–50]. We developed a database to collect variables (described in S2 Appendix) from each of those papers, which yielded a total of 20 effect size estimates (see Results).

### Study quality and moderator classification

One of the critical assumptions in a meta-analysis concerns the quality of the studies included. Assessing study quality can help account for between-study heterogeneity due to study characteristics [51]. We collected several criteria related to study quality and experimental design (S2 Appendix).

We assessed whether the variation between studies could be accounted for by different covariates via moderator analyses [52], presented below. For each of the nine moderators used in our analysis, we established their levels based on the structure of the dataset we collected, leading to the elimination of some levels (e.g., for temporal matching, no included studies had an incomplete temporal overlap >50%, rendering an “intermediate” level obsolete).

1 **Migratory behavior (3-levels)**

*Migratory*: all species included in the study were considered to be migratory.

*Non-migratory*: all species included in the study were considered to be non-migratory.

*Migratory and non-migratory*: multiple species were studied, where some were migratory, and some were non-migratory. This moderator level was often the best fit for studies collecting data on all species in an airport environment.

We collected this information based on descriptions in the articles; however, when this information was not provided, we consulted Birds of the World [53] or species-specific articles, cited in S2 Appendix, to establish the migratory status (and flocking behavior, see below) in the region of the studied airport. We excluded from this moderator analysis articles that did not identify the species included (category “unclear” in S2 Appendix).

2 **Flocking behavior (3-levels)**

*Flocking*: all species included in the study formed conspecific flocks.

*Non-flocking*: all species included in the study did not typically form flocks.

*Flocking and non-flocking*: multiple species were studied, where some were prone to flocking, and others were not. This moderator level was often the best fit for studies collecting data on all species in an airport environment.

This information was collected from the primary article when possible, and outside sources (see 1. Migratory behavior) when necessary.

3 **Species level (2-levels)**

*Within-species*: study or subset of study focused on the abundance and strikes of a single species.

*Between-species*: study or subset of study focused on the abundance and strikes of multiple species.

4 **Degree of spatial resolution (2-levels)**

*Within airport*: study focused on bird abundance and number of strikes within a single airport.

*Between airports*: study focused on bird abundance and number of strikes across multiple airports, or across a region.

5 **Temporal matching (2-levels)**

*High match*: bird abundance and bird strike data were collected over the exact same period of time.

*Low/No match*: bird abundance and bird strike data were collected over different periods of time, where there was less than a 50% overlap in the time periods each dataset was collected. No included studies had an incomplete temporal overlap > 50%. Generally, this overlap or lack thereof was measured in months. Temporal bins (i.e., days, weeks, months) were, however, limited by the sampling period provided in the original study.

6 **Spatial matching (2-levels)**

*High match*: bird abundance and bird strike data were collected over exactly the same spatial range (i.e., within the boundary of an airport).

*Intermediate match*: bird abundance and bird strike data were not collected over exactly the same spatial range (i.e., abundance data comes from areas under approach or departure airspace).

No studies had a complete lack of overlap in the spatial range of bird abundance and frequency of strike data.

7 **Bird abundance survey method (5-levels)**

*Point count*: surveyor counted birds from a stationary location for a fixed amount of time.

*Transect*: surveyor traveled along a transect and counted birds within a set distance of the transect.

*Nest count*: number of active nests in a given area was used as a proxy for bird abundance.

*Radar*: flocks of birds which appear on radar were counted.

*Multiple methods*: Bird abundance data were taken from secondary sources (e.g., Waterfowl Breeding Population and Habitat Survey, U.S. Fish and Wildlife Service reports, Partners-in-Flight database, etc.) that used a combination of the survey methods described above.

8 **Bird abundance data source (2-levels)**

*Primary*: direct abundance measurements were made during the study (i.e., researchers performed bird surveys).

*Secondary*: abundance estimates came from previous studies, previous surveys by airport personnel, or external databases.

9 **Bird strike frequency data source (2-levels)**

*Primary*: direct bird strike data was collected during the study (e.g., researchers collected DNA samples following collisions)

*Secondary*: number of bird strikes reported came from previous studies or external databases.

We chose not to include habitat type as a moderator due to the challenges of classifying diverse habitat types over large areas. In some cases, studies spanned entire countries and could not be categorized into few enough levels to be statistically analyzed.

### Effect size computation

We focused on the association between bird abundance and the frequency of bird strikes. Thus, we selected Pearson’s correlation coefficient (*r*) as a measure of effect size [54]. We followed Quintana’s [55] recommendations for meta-analysis examining correlation analyses: for studies that did not report Pearson’s *r*, we calculated effect size(s) from provided datasets. If the raw data were not included, we estimated Pearson’s *r* values from included figures using WebPlotDigitizer (https://automeris.io/WebPlotDigitizer/), which allowed us to scan and extract data points. After obtaining bird abundance data and strike frequency data, we calculated Pearson’s *r* as our effect size for each outcome [38] as:


$$\begin{array}{c} =\frac{\sum_{i}(Xi-\overset{―}{X})(Yi-\overset{―}{Y})}{\sqrt{\sum_{i}{(Xi-\overset{―}{X})}^{\text{2}}}\sqrt{\sum_{i}{(Yi-\overset{―}{Y})}^{\text{2}}}}; \end{array}$$

where for each *i* observation, x_i_ is the i^th^ abundance, and y_i_ is the i^th^ strike frequency. An *r* = 1 represents a perfect positive association between variables, *r* = −1 is a perfect negative association, and *r* = 0 indicates no association between variables. The standard error of a correlation is a function of not only sample size but also the effect size itself [38]. Estimating the SE in studies with a small sample size could bias the meta-analysis [56]; Pearson’s *r* also has a skewed distribution, especially when dealing with very large effect sizes approaching 1 [57]. Consequently, we converted all effect sizes (Pearson’s *r*) to the Fisher’s *z* metric following:


$$\begin{array}{c} Z=\text{0}.\text{5}\times \text{ln}(\frac{\text{1}+\text{r}}{\text{1}-\text{r}}) \end{array}$$

Sample standard error was similarly recalculated using the formula below.

To calculate the standard error (${\text{SE}}_{Z}$) for Fisher’s *z*, where n is sample size:

${\text{SE}}_{Z}={\left(\frac{\text{1}}{\text{n}-\text{3}}\right)}^{\text{0}.\text{5}}$ [37].

We estimated Pearson’s *r* and Fisher’s *z* effect size for each individual outcome. Some studies produced multiple outcomes (S2 Appendix).

After running the meta-analysis (see below), we converted the pooled effect size and its confidence intervals back to Pearson’s *r* metric for ease of interpretation with the following equation:


$$\begin{array}{c} =\frac{e^{\text{2}Z}-\text{1}}{e^{\text{2}Z}+\text{1}} \end{array}$$

### Meta-analysis

We used a multilevel meta-analytic model in our meta-analysis [58,59]. A multilevel model is most appropriate to analyze this dataset because (1) our studies came from different populations (e.g., different geographic locations, species, etc.), and (2) some papers reported multiple outcomes (i.e., more than one effect size estimate resulting from the comparison of two airports, multiple species, etc.). The multilevel meta-analysis estimates both within-study and between-study variance [60,61]. This model is effectively an extended random-effect meta-analysis that incorporates this nested term (i.e., 1 | Study ID/Outcome ID) in its random effect structure [54, 58]. In our models, we used the restricted maximum-likelihood (REML) estimator [54].

We estimated the heterogeneity in our pooled effect size, which represents the variation in study outcomes (i.e., observed effect sizes) among studies. We further break this heterogeneity into variation resulting from between-study variance and within-study variance, to account for those studies producing multiple effect sizes. Our multilevel meta-analysis considered from each outcome the observed Fisher’s *z* effect size, the variance of each observed effect size, and the identity of the study [62]. Our primary multilevel meta-analytic model was an intercept-only model, with study identity and outcome identity (i.e., the identity of a single effect size) included in the random structure as nested effects (i.e., identity of the outcome nested within the identity of the study) following Harrer et al. [54]. The pooled effect size was considered statistically significant if the *P*-value < 0.05, indicating that an association between bird abundance and strike frequency exists (i.e., the confidence interval of the pooled effect does not overlap zero). We present the pooled effect size with the associated 95% confidence intervals. Each effect was weighted by the standard error of its Fisher’s *z* score, which essentially reflects its sample size (i.e., higher sample sizes have higher weights).

We assessed the heterogeneity in our meta-analysis as the variation in study outcomes (observed effect sizes) between studies [54]. For a given sample size, high heterogeneity reduces statistical power, particularly for estimates of moderator effects (see below). We used two metrics of heterogeneity in this meta-analysis: the Cochran’s *Q*-statistic and the *I^2^*, which is an estimate of the variation in effect sizes between studies not attributable to sampling variance [37,54,63]. Cochran’s *Q*-statistic can be used to test the null hypothesis that no heterogeneity is present, with a statistically significant *P*-value indicating the presence of heterogeneity among different effect sizes [63]:


$$\begin{array}{c} Q=\sum \overset{K}{\underset{K=\text{1}}{\mathrm{\backslash nolimits}}}\omega_{k}{\left(\hat{\theta_{k}}-\hat{\theta }\right)}^{\text{2}}; \end{array}$$

where $\hat{\theta_{\text{k}}}$ is the observed effect size of each study, $\hat{\theta }$ is the pooled effect size, and $\omega_{\text{k}}$ is the inverse of the study variance. We also calculated the *I^2^* statistic as [54]:


$$\begin{array}{c} I^{\text{2}}=\;\frac{Q\;-\;(K\;-\;\text{1})}{Q}; \end{array}$$

where K is the total number of studies in the meta-analysis, *Q* is Cochran’s *Q*-statistic. We report total heterogeneity, as well as its summed parts, between-study and within-study variance [64].

We investigated potential sources of heterogeneity by running moderator analyses, or subgroup analyses (i.e., a multilevel meta-analytic model with a categorical moderator of interest included as a fixed effect), which allowed us to assess which, if any, covariate factors might significantly influence the pooled effect size estimate or contribute to observed heterogeneity. These subgroup analyses pool studies within categorical moderator levels to establish whether pooled effect size estimates differ significantly between levels. We ran individual subgroup analyses for each of the moderators described above instead of including multiple moderators in a single analysis. We acknowledge this was not an ideal approach, but in a meta-analysis with relatively low sample sizes, including all moderators in a single analysis can skew results and prevent inclusion of interaction effects [52,65,66]. Therefore, these analyses were not fully independent. These analyses generated two *P*-values, one from a *Q*-test for the model that assessed whether heterogeneity exists among the effect size estimates within each moderator level, and one coming from the analysis of variance for the residuals of the model that determines whether effect size estimates among moderator levels are different from each other (i.e., investigating whether the single moderator significantly influenced pooled effect size). Additionally, moderator effects on heterogeneity are investigated via the marginal *R*^2^ value of the moderator-included meta-analytic model [58]. We subsequently ran the model with the intercept removed to assess whether the mean effect size and confidence intervals of each level within the moderator were significantly different from zero (i.e., confidence intervals not including zero; [52]).

Because of publication bias (e.g., publishing only significant results), studies reporting large effect sizes are more likely to be present in the literature than studies reporting smaller effect sizes [67–69]. Publication bias, if present, might result in an overestimation of the pooled effect size relative to the true population-level effect size. We assessed the potential for publication bias using funnel plots, in which 95% of included studies should remain within the bounds of the funnel (representing the 95% confidence intervals). We used three types of funnel plots to visualize and interpret our results: the classic funnel plot, a three-level funnel plot [70], and a contoured funnel plot [71]. In a three-level funnel plot, mean effect size estimates for each study are plotted; size of the point corresponds to the number of individual outcomes in each study. We additionally conducted an Egger’s test [72] to quantify whether asymmetry existed in our funnel plot. If the observed effect sizes are distributed asymmetrically around the mean effect size (e.g., several effect sizes located in the lower-right part of the funnel but none with a similar imprecision located on the funnel’s lower-left), it could suggest the presence of publication bias [73].

Although funnel plot asymmetry is often used as evidence of publication bias, the asymmetry could be also attributed to other factors (e.g., differential study quality, heterogeneity; [74,75]). Therefore, we also generated a contour-enhanced funnel plot to distinguish publication bias from other forms of asymmetry [71]. Different shades in contour-enhanced funnel plots represent different significance levels (set as α-levels of 0.90, 0.95, and 0.99, equivalent to *P* < 0.1, 0.05, and 0.01, respectively). If studies appear to be missing in areas of low statistical significance (*P* > 0.05), the asymmetry of a contour-enhanced funnel plot could be attributed to publication bias; whereas asymmetry is less likely to be caused by publication bias if studies appear to be missing in areas of high statistical significance (*P* < 0.05; [74]).

Finally, we investigated the effects of studies with small sample sizes on our pooled effect size, using a test for small-study effects [76]. This test is conducted as a moderator analysis, where each outcome standard error is fit into a meta-regression (i.e., moderator analysis of a continuous variable) of the multilevel model as a fixed effect. If results are significant, this test indicates that small-study effects significantly affect the pooled effect size. Like the subgroup analyses described above, we also investigated the proportion of total heterogeneity attributable to small-study effects.

We estimated the power of the studies included in our meta-analysis using firepower plots, in which study power is estimated from the study effect size(s) and standard error [77]. For this analysis, α is assumed to be 0.05. Firepower plots provide the median statistical power for a range of effect sizes, assuming that pooled effect sizes are indeed the true effect [77]. We present two firepower plots: one displaying the estimated statistical power of the pooled effect size in our meta-analysis, and another displaying the estimated statistical power for each individual study included in our meta-analysis. For the latter firepower plot, we assumed that each individual study was a “mini meta-analysis” [77]. Firepower plots provide an indication of whether included studies are under- or sufficiently-powered to detect relevant effect sizes [77,78].

We used R [79] to run our analyses. To calculate Pearson’s *r* values, we used the function “cor.test()”in base R. Meta-analysis and moderator analyses were conducted using the “rma.mv()” function, and basic funnel plot was constructed using the “funnel” function, both from the “metafor” package [80]. Traditional forest plot was constructed using modified code originally published by Fernández-Castilla et al. [70]. Firepower plots were developed with the “metameta” package [77]. Heterogeneity and visualization of moderator analyses were done using the “orchard 2.0” package with “i2_ml()” and “orchard_plot()” functions, respectively [81].

## Results

**Fig 2:**
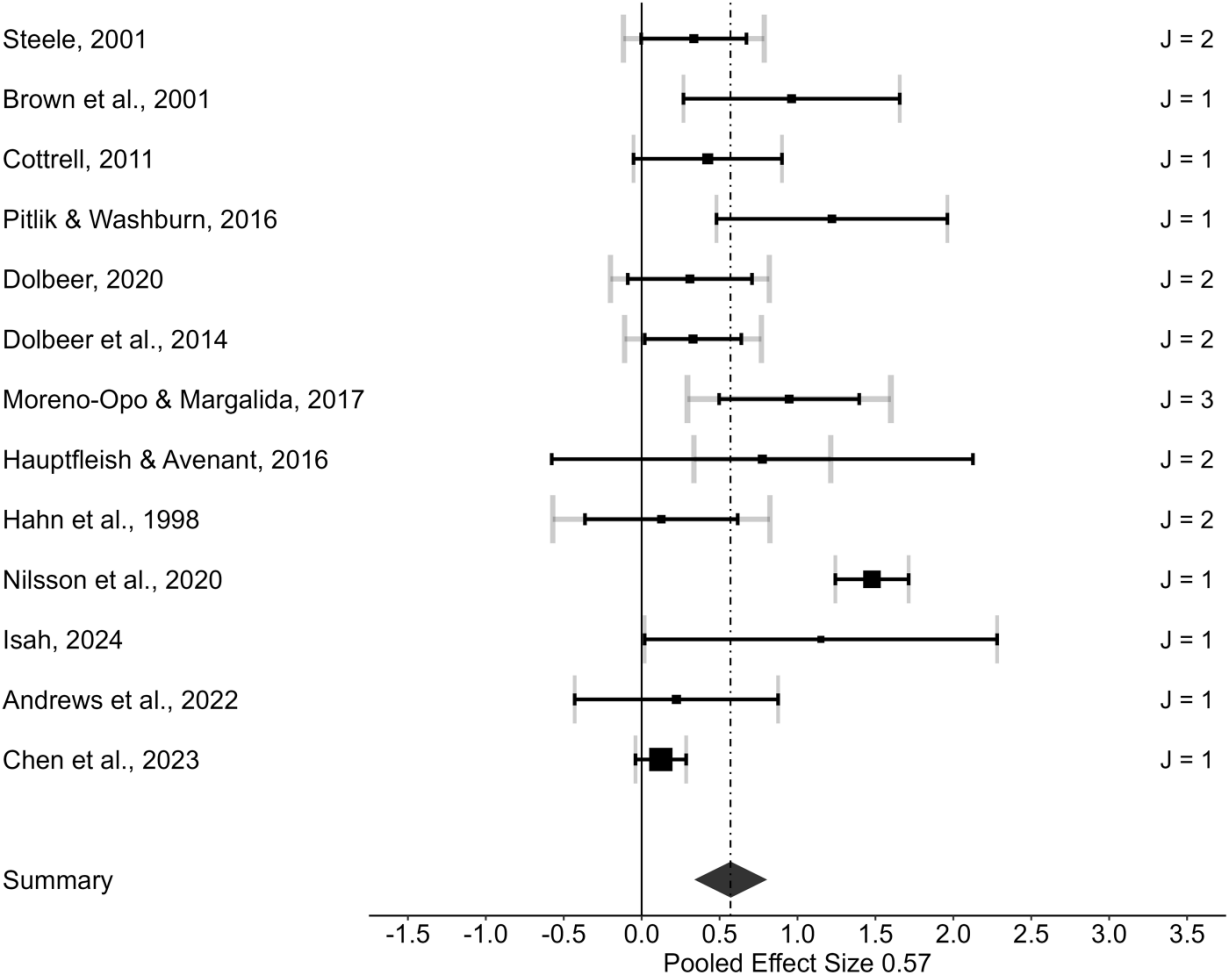
Forest plot of multilevel meta-analysis. J = number of effect sizes from each study. Each study’s estimated effect size (Fisher’s *z*) is represented by a black square whose size is proportional to the weight of the study in the meta-analysis. Black horizontal lines indicate the 95% confidence interval (CI) across study outcomes, and grey horizontal lines (95% CI) illustrate the median precision of a single effect size within the study. The pooled effect size estimate is plotted as a black diamond at the bottom of the forest plot, with the width of the diamond being the pooled 95% CI across all effect size estimates. When a confidence interval (black lines) crosses zero, the effect size is non-significant (i.e., no different from zero).

Of the 378 studies we screened, we identified 13 that met our inclusion criteria (Fig 1; Table 1; [27–30,42–50]). Six of thirteen studies reported multiple outcomes (more than one effect size; Table 1). Overall, we obtained 20 outcomes from these studies. After standardizing the effect sizes to Pearson’s *r* (range: −0.113–0.949, Table 1), all outcomes were included in our meta-analysis (K = 20). Sample sizes for individual effects ranged from 4 to 149 (Table 1). All but one outcome showed a positive Pearson’s *r*, although only nine of those were significantly different from zero (Table 1). Based on study confidence intervals, eight of the thirteen studies showed no significant correlation between bird abundance and strike frequency (i.e., confidence intervals crossed zero, Fig 2). Several studies that produced multiple effect sizes had variable effect size estimates across outcomes. This is indicated in Fig 2 by total precision across outcomes (black bars) being higher than precision per outcome (gray bars; indicative of highly variable within-study effects, e.g., [46]), and vice versa (often resulting from low sample sizes, e.g., [30]).

**Table 1:** Statistical data from 13 studies and 20 outcomes obtained in our meta-analysis. Sample size reflects the number of samples after extracting data and removing species/temporal units with 0 values for abundance. Pearson’s *r* is a) the effect size estimate from the study or b) calculated effect size based on data extracted from figures or tables (indicated in superscript). *P*-values are from correlation tests. Fisher’s *z* is Pearson’s *r* transformed using the equations provided in the methods section. SE values associated with each Fisher’s *z* were calculated using the equation provided in the methods section.

Reference	Study ID	Outcome ID	Sample size	Pearson’s *r*	*P*-value	Fisher’s *z*	SE_z_
Steele, 2001	1	1	12	0.013^b^	0.968	0.013	0.333
Steele, 2001	1	2	64	0.402^b^	0.001	0.427	0.128
Brown et. al, 2001	2	3	11	0.745^b^	0.009	0.962	0.354
Cottrell, 2011	3	4	20	0.4^b^	0.08	0.424	0.243
Pitlik and Washburn, 2016	4	5	10	0.84^a^	0.003	1.221	0.378
Dolbeer, 2020	5	6	20	0.456^b^	0.043	0.492	0.243
Dolbeer, 2020	5	7	16	0.083^b^	0.761	0.083	0.277
Dolbeer et. al, 2014	6	8	23	0.211^b^	0.333	0.215	0.224
Dolbeer et. al, 2014	6	9	23	0.417^b^	0.048	0.444	0.224
Moreno-Opo & Margalida, 2017	7	10	12	0.787^b^	0.001	1.064	0.333
Moreno-Opo & Margalida, 2017	7	11	12	0.624^b^	0.028	0.732	0.333
Moreno-Opo & Margalida, 2017	7	12	4	0.949^b^	0.047	1.822	1.000
Hauptfleish & Avenant, 2016	8	13	22	0.083^b^	0.721	0.083	0.229
Hauptfleish & Avenant, 2016	8	14	24	0.898^b^	<0.001	1.462	0.219
Hahn et. al, 1998	9	15	12	0.302^b^	0.34	0.312	0.333
Hahn et. al, 1998	9	16	10	−0.113^b^	0.757	−0.113	0.378
Nilsson et. al, 2020	10	17	73	0.901^b^	<0.001	1.478	0.120
Isah, 2024	11	18	8	0.819^a^	0.013	1.15	0.577
Andrews et al. 2022	12	19	12	0.22^b^	0.493	0.223	0.333
Chen et al. 2023	13	20	149	0.122^b^	0.138	0.123	0.083

Our assessment of study quality provided some insight into the constraints studies had in terms of experimental design (S2 Appendix). Despite the different methods used in the bird surveys (transects, point counts, nest counts, etc.), none of the studies reported an assessment of the independence of their sampling units. Similarly, there was no report in any studies on the degree of independence in bird strike frequency measurements across sampling areas within airports. Additionally, none of the studies reported conducting an *a-priori* power analysis to estimate the sample size required to detect a given effect size. Regarding the degree of temporal matching between the bird abundance and bird strike frequency data, five studies showed <50% overlap in collection dates of abundance and strike data or, in one case, no match. Six studies had intermediate spatial matching, meaning abundance data was collected outside airport boundaries. There was also an unbalanced geographical distribution of the studies in our dataset. North American studies made up the majority (n = 7), which two each from Australia and Europe, and one each from Africa (Namibia) and Asia (China).

Our multilevel meta-analysis indicated an overall significant and positive correlation between bird abundance and strike frequency (*t*_20_ = 4.68, d.f. = 19, *P* = 0.002). The pooled effect size was estimated as Fisher’s *z* = 0.576, with 95% confidence intervals (0.318, 0.834; Fig 2) that did not cross zero. After transforming the pooled Fisher’s *z* to Pearson’s *r*, the pooled effect size was estimated as *r* = 0.520, with 95% confidence intervals = 0.308, 0.683. From these results, the pooled effect size estimate was significantly different from zero and positive. In other words, an increase in bird abundance was associated with an increase in bird strike frequency.

Our Cochran’s *Q* test was significant (*Q* = 132.49, d.f. = 19, *P* < 0.001), indicating that heterogeneity existed within or between studies. Our total *I^2^* (variation not attributable to sampling variance; [58]) was 81.07%. Study identity and study identity/outcome identity had *I^2^* values of 8.28% and 72.79%, respectively.

**Fig 3:**
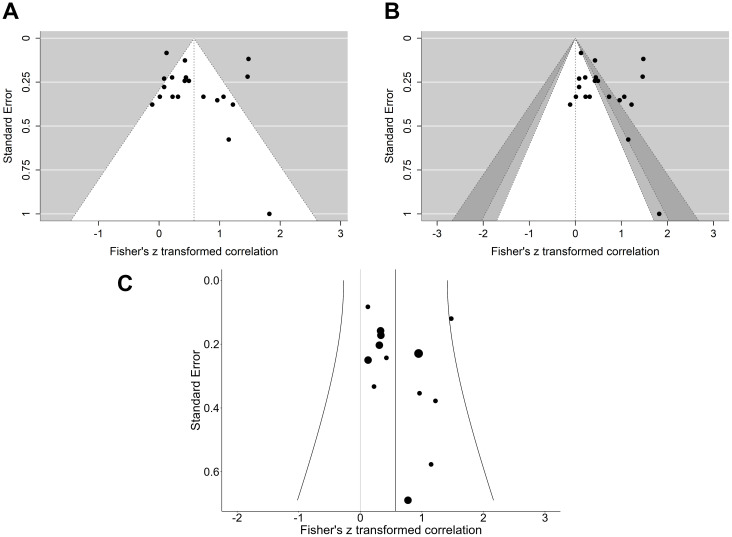
Traditional and three-level funnel plots, where the y-axis is Fisher’s transformed standard error (transformation of effect sample size). **(a)** Traditional funnel plot with each point representing an effect size outcome. The borders of the funnel are 95% confidence intervals, and consequently 95% of the effect size estimates are expected to be located within the funnel, given no publication bias. **(b)** Contour-enhanced funnel plot, where the effect size estimate is centered around zero. White, light gray, and dark gray bounds of the funnel correspond to *P*-values of >0.1, 0.1 > 0.05, and 0.05 > 0.01, respectively. Effects outside the funnel have *P*-values < 0.01. **(c)** Three-level funnel plot, with each point representing a study. The size of points corresponds to the number of effect sizes included in that study.

All three of our constructed funnel plots (Fig 3) displayed some degree of asymmetry. The results from our Egger’s test also indicated asymmetry (Intercept = 0.462, 95% confidence interval = 0.144, 0.780, *t* = 3.049, *P* = 0.007). Of note, there were few studies near the bottom of the funnel (Fig 3a), suggesting a lack of reported estimates with low precision. The contour-enhanced funnel plot (Fig 3c) suggested missing non-significant effect size estimates (*P* > 0.05), as well as an abundance of highly significant findings outside the funnel. Between the asymmetries and irregular, scattered placement of the effect size estimates, our results indicate a clear presence of publication bias in this literature. There was no significant effect on the pooled effect size from small-study effects (*F*_1,18_ = 0.759, *P* = 0.395), suggesting that studies with small sample sizes did not affect the overall effect size. However, small-study effects accounted for 10.6% (*R*^2^) of overall heterogeneity.

We conducted subgroup analyses for each of our moderators using the same structure as our multilevel model, except with the moderator of interest included as a fixed effect. These tests only assess the effect within a single moderator. None of the moderator analyses yielded significant results, indicating no statistically significant differences exist between moderator categories (Table 2). However, several of our moderators were shown to contribute to our meta-analysis heterogeneity. The moderators with the highest *R*^2^ values (percent heterogeneity explained) included source of bird strike frequency data (23.9%), abundance survey method (11.9%), and source of bird abundance data (8.7%; Table 2).

**Table 2:** Results of moderator analyses including the *F* statistic, degrees of freedom (d.f.), and *P*-values to assess the difference between moderator levels in categorical moderators. Also shown are the results of Cochran’s *Q* tests for between-study heterogeneity, including *Q* statistic, degrees of freedom (d.f.), and *P*-value, and *R*^2^ values associated with the subgroup analyses of each moderator.

Moderator	*F*	d.f.	*P*-value	*Q*	d.f.	*P*-value	*R*^2^ Value
Bird abundance survey method	0.739	2, 15	0.494	54.369	15	< 0.001	0.119
Migratory behavior	0.321	1, 15	0.580	127.433	15	< 0.001	0.029
Bird strike frequency data source	3.852	1, 18	0.065	128.665	18	< 0.001	0.239
Temporal matching	0.450	1, 16	0.512	92.927	16	< 0.001	0.038
Flocking behavior	0.213	2, 15	0.810	127.105	15	< 0.001	0.036
Bird abundance data source	1.227	1, 18	0.283	129.325	18	< 0.001	0.087
Species level	0.233	1, 18	0.635	132.278	18	< 0.001	0.018
Spatial matching	0.069	1, 18	0.796	132.295	18	< 0.001	0.005
Degree of spatial resolution	0.001	1, 18	0.972	107.527	18	< 0.001	0.010

**Fig 4:**
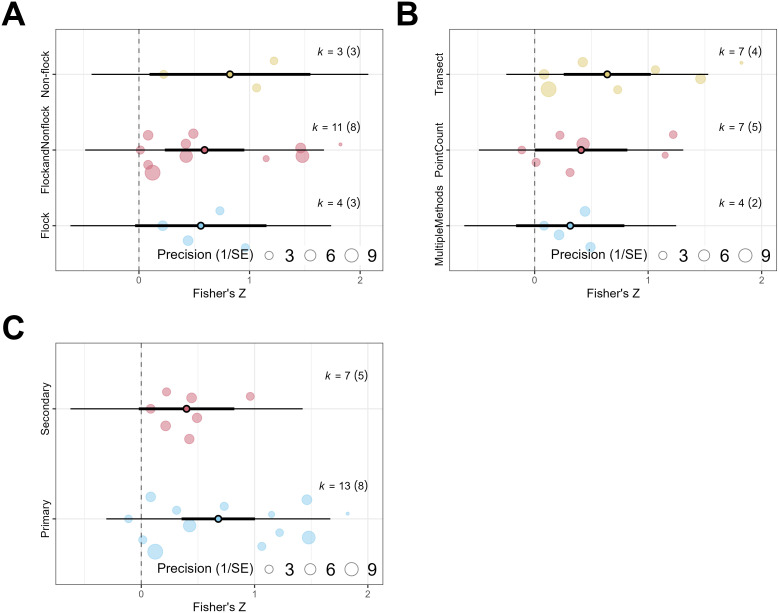
Comparisons of pooled effect size across moderator levels for moderators which had effect size confidence intervals cross zero. **(a)** Flocking behavior moderator, **(b)** Abundance sampling method, **(c)** Source of abundance data. Bolded black bars indicate a 95% confidence interval around the pooled effect size; thin black bars indicate prediction intervals. Individual circles represent each outcome in the moderator analysis, with size corresponding to that outcome’s precision (1/SE).

We then examined whether coefficient estimates for the different levels in each intercept-removed moderator analysis were different from zero via an inspection of their 95% confidence intervals. The significance levels of the moderator analyses play no role in whether individual factors have confidence intervals which overlap zero. All moderator-level effect sizes and their associated 95% confidence intervals can be found in S1 Table. For migratory behavior, the effect size estimate for both non-migratory species and the combination of migratory and non-migratory species were positive and different from zero. Only one study ([29]; Fig 2) examined exclusively migratory birds and was not included in this moderator analysis to avoid category sample sizes of 1. For flocking behavior, the effect size estimates for both non-flocking species and for studies that included both flocking and non-flocking species were different from zero, but effect size for flocking species only was no different from zero. This suggests a positive relationship between bird abundance and strike frequency in solitary species (Fig 4a). However, both flocking (n = 5) and non-flocking (n = 3) categories had low sample sizes and large associated variance. For species level, degree of spatial resolution, temporal matching, and spatial matching moderators, the effect size estimates for all moderator levels were positive and different from zero.

For abundance survey method, only three of five methods could be compared (nest counting and radar counting were used in one study each). The effect size estimates for studies that used transects and point counts were both positive and different from zero, but the effect size estimate for studies that used multiple methods was no different from zero (Fig 4b). For abundance data source, the effect size estimate for studies that used primary data sources was positive and different from zero, but for studies that used secondary data sources, the effect size estimate was no different than zero (Fig 4c). For bird strike frequency data source, the effect size estimates for studies that used primary and secondary data sources were both positive and different from zero.

**Fig 5:**
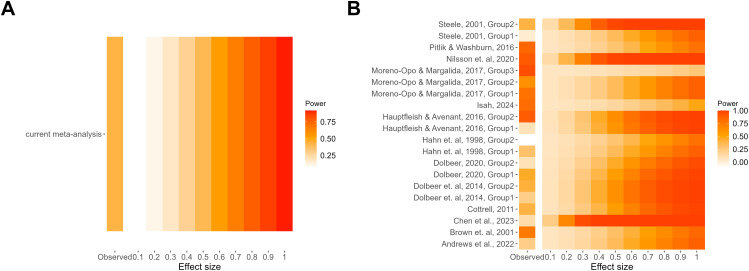
Firepower plots displaying statistical power for a range of possible “true” effect sizes. Higher statistical power is associated with higher saturation. The x-axis displays hypothetical effect sizes (0.1–1.0), corresponding to the power needed to detect them. Plot (a) indicates power for our meta-analysis (α = 0.05, n = 20). Plot (b) shows the power of the individual studies included in our meta-analysis (α = 0.05, n = values reported in Table 1).

Finally, we estimated the statistical power of the studies in our dataset using firepower plots. Fig 5a indicates the median observed power of our meta-analysis was 42.6%, assuming the pooled effect size (Pearson’s *r* = 0.520) is the true effect size of this system, and the median power for individual studies is shown in Fig 5b. This finding indicates that the literature sampled for this meta-analysis is underpowered as a whole. Therefore, our effect size estimations would allow us to reliably detect a significant relationship between bird abundance and strike frequency with 80% power only when effect sizes are large (Pearson’s *r* > 0.90).

## Discussion

Wildlife management at airports is based, generally, on the tenet that animal abundance correlates positively with strike frequency. Understanding that airport wildlife biologists cannot necessarily wait for additional studies to be conducted and published, we can suggest that our meta-analysis (based on the literature surveyed) supports this assumption, but with caveats. Only thirteen studies (20 effect sizes) met the inclusion criteria of our meta-analysis, which suggests that empirical tests of the abundance-strike prediction have been rather limited. This was unexpected given the degree of acceptance of this prediction in the airport wildlife management community. However, our choice to include only English-language studies likely decreased the number of potential studies available for our dataset [82] and may have resulted in English-speaking regions being overrepresented in our dataset. There is a clear geographic bias in our dataset, with more than 50% of included studies conducted in the United States or Canada; 69% of included studies came from English-speaking countries, adding Australia. Using only English-language studies may also overlook research and management practices published from non-Anglophone regions, which are typically underrepresented in systematic reviews [83]. While there is nothing to indicate the abundance-strike prediction should vary geographically, our results may not be globally generalizable. Biases also likely arise at the study level, with inconsistent abundance sampling methods and incomplete strike reports (see below). Our meta-analysis also provided further insights that question the strength of the overall effect size based on the high levels of heterogeneity found between studies and the evidence of publication bias in existing literature.

Our Pearson’s *r* pooled effect size was 0.520, which is considered a large effect size by Cohen’s conventions [84]. With so few studies, we suspect this effect size could be artificially inflated by high-powered studies reporting large effects (e.g., [47]). Nevertheless, these studies corroborate the expected pattern. Also note the relatively low power achieved across our included studies (Fig 5), which indicates correlations taken from these studies should be interpreted with caution. The confidence interval around this pooled effect size was very large, suggesting low precision (high uncertainty) in the effect size estimate (Fig 2). Effect sizes varied considerably, with seven studies having confidence intervals overlapping zero. Our estimates of heterogeneity confirmed this trend of high between-study variation. Two tests (Cochran’s *Q* and *I*^2^) confirmed the high heterogeneity between effect size estimates.

We investigated, via moderator analyses, whether the high levels of heterogeneity in our meta-analysis might be related to different covariates. None of these moderator analyses provided significant results, (i.e., no significant difference between/among the moderator levels). To maximize our sample size, we included studies that examined the abundance-strike prediction at the within-species/guild level and between-species level (S2 Appendix) and sample sizes varied by an order of magnitude across studies (S2 Appendix). This high level of variation in sampling unit, temporal/spatial level of analysis, etc. might have introduced enough noise to reduce our chances of detecting significant moderator effects. It is possible that the apparent homogeneity across moderator levels results from the low observed power of the included studies (Fig 5) and the high between-study heterogeneity. However, it is not possible to parse out the relative importance of these factors.

However, we identified the relative contribution of each moderator, as well as small-study effects to overall heterogeneity (marginal *R*^2^). Between tested moderators and small-study effects, more than half of total heterogeneity can be accounted for. The remaining heterogeneity is likely due to publication bias, or to additional study- and species-specific considerations that could not be detected in the moderator analysis (i.e., discrepancy between measured abundance and actual abundance, population-level habituation to aircraft, fine-scale habitat characteristics, etc.). The air traffic density of included airports may also play a role – large airports with high traffic densities might incur higher numbers of bird strikes simply because there are more opportunities for a bird strike to occur, even if bird abundance levels were similar to a smaller airport. While we were unable to directly compare air traffic density in this meta-analysis, this variable warrants future investigation.

Our moderator analysis also allowed us to establish the levels at which effect size estimates were different from zero, indicating that the association between bird abundance and strike frequency was significant in that particular category. For instance, the abundance-strike prediction was supported in studies on both resident species and combined migratory/resident groups, and on non-flocking species. To lower associated variance in future analyses of the abundance-strike relationship, well-designed surveys should consider pulses of migrants [85], particularly at night [47], because strikes within the approach/departure corridors (e.g., ≤ 1500 ft Above Ground Level; [8]) might involve migrating individuals not detected in standard airport surveys (e.g., [86]). The comparison across flocking behavior yielded results indicating that the abundance of flocking species does not appear to affect the frequency of strikes. This finding might result from the majority of strikes with common flocking species occurring with single individuals (e.g., Canada Goose, 60.3%; European Starling *Sturnus vulgaris*, 70.3%; [10]). Experimental design moderators relating to the spatial and temporal matching of abundance and strike data showed no difference between levels, suggesting that, within reason, historical abundance and strike data may be compared to test the abundance-strike hypothesis. Regarding strike data, studies collecting original data (primary) and those using secondary data (e.g., from the FAA Wildlife Strike database) both supported the abundance-strike prediction. This is a promising result, given that the collection of original strike data can be prohibitive for researchers without direct access to an airport. However, note the large proportion of heterogeneity attributable to this moderator (23.85%). The moderator analysis for abundance data indicated that only primary collections of abundance data supported the association between abundance and strike frequency. The observed effect size for studies taking abundance data from previous datasets was no different than zero. Finally, of the three abundance survey methods assessed, both point counts and line-transect survey methods supported the abundance-strike prediction.

Quantifying species’ relative abundance and associated variation on airports demands objective, bias-corrected surveys standardized relative to diurnal period and seasonal frequency, which is not necessarily the case currently [85]. Buckland [87] suggested that line-transect surveys were more efficient than point-transect surveys for songbirds. However, airport surveys rarely include aurally detected individuals (because of high levels of ambient noise), do not often consider species “hawking” over land cover and those loafing on structures, and survey points are sometimes fixed-radius sample stations [85,86]. Blackwell et al. [85] suggest that point-transect surveys should be used, with points systematically located along transects, which should be uniform and predetermined (i.e., transects 400 m apart, with 400-m separation between points on the transect) and sampled randomly, maintaining stratification.

One reason behind the remaining unexplained heterogeneity in effect size estimates could be related to publication bias. The asymmetries we found across funnel plots (particularly the contour-enhanced funnel plot) suggest the existence of publication bias in the papers published on the abundance-strike prediction. Additionally, the bottom portion of the funnel plot (low precision) had very little representation (only one effect size) across the range of expected effect sizes (Fig 3). This finding might be the result of studies with smaller effect sizes or with small sample sizes having been not submitted or rejected from journals. Moreover, two of our largest effect sizes came from studies with very low variation; these studies might be pulling the pooled effect farther into the positive range. Overall, there appears to be a bias towards publishing studies with large effect sizes and against reporting results of studies with relatively low precision.

### Implications for future tests of the abundance-strike prediction

One of the implications of our meta-analysis is that more empirical tests are necessary to thoroughly evaluate the abundance-strike prediction and more accurately estimate its pooled effect size. We propose several methodological suggestions to improve future testing of the association between bird abundance and strike frequency.

First, the description of the methods in included studies varied to such a degree that some studies would not be reproducible with the limited information provided (e.g., no reporting on the width of bird sampling strips, distance between sampling plots/transects, independence of sampling units). It is especially important that future studies clearly describe the parameters used to ensure independence of replicates. Additionally, many studies did not report testing key assumptions of avian surveys (e.g., detection bias; [85,88]). At the between-species scale, many studies implicitly assumed that the probability of detecting individuals from different species is the same, which is unlikely given interspecific differences in ecology and behavior. At the within-species level, we also found the implicit assumption that individuals were equally likely to be detected in different habitat types, times of the day, etc., which is unrealistic. Many papers did not report testing the assumptions of their statistical analyses (i.e., independence of data) or for outliers.

Second, some tests in the included studies at the between-species level apparently considered data in which a given species had zero abundance and zero bird strikes (or zero reported abundance and a non-zero number of reported strikes); this approach cannot logically be included in the statistical analyses. Zero-based data should be considered with caution in future tests. If a species has not been detected (i.e., zero abundance) in surveys, but bird strikes on that species have been reported, it might indicate an issue with the sensitivity of the survey technique used, if not explainable by species-specific seasonal movements.

Third, it is essential that all future studies with a sound design are published (even if the statistical results are non-significant) to avoid publication bias, and that raw data are made available for future meta-analyses. These studies should consider the logistical differences of using within-species vs. between-species data to test this prediction. Within-species data collection requires extensive sampling effort across different portions of the airport (within-airport level) or across different airports (between-airport level) to satisfy sufficient levels of replication (see above) over a given period. Within-species data would be appropriate in cases of species with high strike risk (e.g., Canada Geese; [5]) or species of conservation concern (e.g., Golden Eagles *Aquila chrysaetos*). Based on our results, within-species data have a high chance of detecting the abundance-strike correlation, which could be particularly relevant if airports have species-specific targets for management (note, however, that we failed to detect a significant correlation for flocking species).

Another challenge is gathering bird strike data. The most extensive open-access database for wildlife strikes is held by the Federal Aviation Administration (FAA) in the United States (https://wildlife.faa.gov/). We are not aware of open-access, nationwide datasets in other parts of the world that receive comparable, regular reporting of wildlife strike data, including aspects of species involved, aircraft, phase of flight, location, and level of damage. Supporting international efforts to build similar open-access datasets in other regions would help to assess the validity of the abundance-strike prediction in airports across the world. Moreover, we encourage pilots and airlines to report any and all wildlife strikes, in line with guidance provided in the International Civil Aviation Organization Document 9137 [20] (see also [89,90] for discussion of bias introduced by pilot non-reporting). Where feasible, all bird strike residue should also be DNA-tested, to ensure strikes are attributed to the correct species and reduce reporting biases for larger species whose carcasses are more easily recoverable [50,91–93].

One of the advantages of our meta-analysis is that it informs sample size decisions for future tests to minimize both Type I (i.e., concluding that the prediction is supported, when it is not) and Type II (i.e., concluding that the prediction is not supported, when it is) errors. Our pooled effect size estimates can be used to calculate sample size for future power analyses. For instance, for a within-species study, using the lower 95% confidence interval limit (all values converted to Pearson’s *r*; 0.206), the mean effect size (0.580), and the upper 95% confidence interval limit (0.806) of the within-species moderator level (S1 Table), future studies would need 300, 32, and 13 sampling units, respectively, for such a study to minimize both Type I and II errors to 5% (α = 0.05, Power = 0.95; calculations with R package “pwr” command ‘pwr.r.test()’, with two-sided analysis; [94]). To contextualize within the existing literature, studies summarized in Table 1 had sample sizes varying between 4–149 sampling units.

## Conclusions

This meta-analysis provides the first synthesis supporting a tenet long-assumed to be true by airport biologists and wildlife managers – with more birds, there will be more strikes. Based on these results, we can provide three specific recommendations. First, wildlife managers should recognize that the number of birds in the airport environment imposes direct safety hazards. Second, airport managers should employ non-lethal and lethal methods to reduce the numbers of birds in the airport environment but should do so in the context of their local survey information and past strikes. Third, given the relevance of having accurate bird density data, managers should regularly conduct surveys following methodologies that fit the airport environment and their workload. However, the low sample size and high heterogeneity of this meta-analysis along with the geographic bias present indicate that our pooled effect size should be treated as a preliminary estimate of the abundance-strike relationship, with a clear need for additional studies using larger sample sizes and the survey practices described above to supplement the literature for future meta-analyses. We identified several shortcomings in the current literature, which may have led to the large variance associated with our results. Consequently, the estimates of this meta-analysis should be taken cautiously while prioritizing the safety of passengers.

## Supporting information

S1 Appendix Search strings and number of results for databases searched. (XLSX)

S2 Appendix Database of identification information, moderator data, and effect sizes for all included studies. (XLSX)

S2 Appendix Metadata Explanation Plain language descriptions of all columns in S2 Appendix. (DOCX)

S1 Table Estimated effect sizes (Fisher’s *z*) and 95% confidence intervals for each level of included moderators. (DOCX)

S1 PRISMA Checklist Line-item checklist of the location in the manuscript of where each requirement of the PRISMA reporting guidelines are addressed. (PDF)
