# Supplementary material for: Do more birds mean more bird-aircraft collisions? A meta-analysis testing a key wildlife management tenet
Source: PLoS One. 2026 Jul 1;21(7):e0349352. doi: 10.1371/journal.pone.0349352 (PMC13322527; doi:10.1371/journal.pone.0349352)
Supplement: S2 Appendix Metadata Explanation — (DOCX) [file pone.0349352.s003.docx]

Appendix S2 contains variables collected from each study included in our meta-analysis, notes on how data was extracted, and naming conventions of files associated with each study.

This Read.Me file provides descriptions for each column in Appendix S2.

**Paper code**: internal code assigned to the study in our database

**Reference**: authorship and publication year of each study

**Study id**: identification number of each study for the meta-analysis

**Outcome id**: identification number of each outcome (effect size) for the meta-analysis

**Relative size of study area:** identifies the relative scale of the study (i.e., airport(s) within a localized vicinity vs. entire countries

**Airport name:** name, airport code, and location of airports from which the outcome was generated

**Degree of spatial resolution of bird abundance data**: whether bird abundance data was collected on the airport(s) of interest, nearby, or estimated from other datasets

**Degree of spatial resolution of strike frequency data**: whether bird strike data collected on the airport(s) of interest, nearby, or estimated from other datasets

**Degree of spatial resolution of each outcome**: reports whether the study compares abundance to strikes within a single airport, between multiple airports, or across regions

**Degree of spatial resolution of each outcome coded in analysis**: Designator of former column for R code, with two responses: 1) “WithinAirport” and 2) “BetweenAirports”.

**Temporal range of the bird abundance data**: dates for which original authors report collection of bird abundance data at their respective airports. **Note:** for Hahn & Weitz (1998), they report that data collection was conducted during 1994 to 1997 in 15 German airports, but the duration of data collection varied across airports, from one year to three years. No detailed information about data collection length for each airport exists in the text.

**Degree of temporal resolution of bird abundance data**: this column identifies whether (A) abundance data were pooled across the entire temporal range (“Data pooled within range”) of the study for the original analysis (i.e., a comparison across multiple species) – corresponds to “PoolRange” in next column. (B) Data were pooled by some temporal unit (e.g., year, month; “Data pooled by time”) for comparison in the original analysis (i.e., a comparison across years) – corresponds to “YearYear” in the next column.

**Temporal range of strike frequency data**: dates for which original authors report collection of bird strike data at their respective airports. See note above regarding Hahn & Weitz (1998).

**Degree of temporal resolution of strike frequency data:** this column identifies whether (A) strike data were pooled across the entire temporal range (“Data pooled within range”) of the study for the original analysis (i.e., a comparison across multiple species) – corresponds to “PoolRange” in next column. (B) Data were pooled by some temporal unit (e.g., year, month; “Data pooled by time”) for comparison in the original analysis (i.e., a comparison across years) – corresponds to “YearYear” in the next column.

Variables related to habitat information

**Habitat information reported in the paper?:** Describes whether enough information regarding local airport habitat was provided in original study to determine whether habitat label should be ‘Open’ or ‘Mixed’ (Reported/Not Reported)

**Google Maps satellite view for the study area:** for studies which did not report habitat information, we identified habitat type using Google Earth. This column identifies the file name corresponding to each airport’s satellite photo.

**Habitat description**: description of how we identified the habitat type for each study. We used several methods: (1) using information reported in the paper, in which case the study is quoted, (2) used Google Maps satellite images to view the habitat of study area, (3) if the study was conducted across a large region, we assumed habitat type would be ‘Mixed.’

**Habitat category**: the habitat type surrounding the airport where sampling took place, with 2 levels: “Open,” and “Mixed” (a combination of open and closed habitats).

**Habitat category coded in analysis:** the code of the habitat type for each outcome used in the analysis, (“mixed”/“open”).

Variables related to study season information

**Study season information reported?:** identifies whether information regarding the season the study took place in was shared in the original study. All studies included in our meta-analysis did report seasonal information, “Reported”

**Study season description**: the temporal frequency of data collection within the original study

**Study season category**: we were interested whether data was collected during local breeding seasons. This column identifies whether the study collected data in the breeding season (“Breeding”), outside the breeding season (“Nonbreeding”), or year-round (“BreedandNonbreeding”)

Variables related to species information

**Migratory behavior reported?**: whether the original study reports on the migratory behavior of bird species involved in the study (Reported/Not reported).

**Migratory behavior description (from paper or other resources)**: how we identified the migratory behavior descriptor for each study (1) using information reported in the paper, (2) searching for primary literature describing whether bird species involved are migratory in respective study areas. For many studies involving multiple species, some species were migratory, and some were resident.

**Migratory behavior category**: the migratory behavior of the bird species studied for each outcome, with 3 levels: migratory, resident (“non-migratory”), or a combination of the two.

**Migratory behavior category coded in analysis:** the code of the migratory behavior category for each outcome used in the analysis, (“Non-migratory”/ “Migratory” / “MigratoryandNonmigratory”).

**Flock behavior reported**?: whether the original study reports on the flocking behavior of bird species involved in the study (Reported/Not reported)

**Flock behavior description (from paper or other resources**): how we identified the flocking behavior information for each study (1) using information reported in the paper, (2) searching for primary literature describing whether bird species involved generally flock. We aimed to describe the species’ typical behavior, acknowledging that some “non-flocking” species like the American Kestrel may rarely been seen in pairs or very small flocks. For many studies involving multiple species, some species flock, and some do not.

**Flock behavior category**: describes whether included species to flock, with 3 levels: flock, non-flock, or a combination of both.

**Flock behavior category coded in analysis:** the code of the flock behavior category for every each outcome used in the analysis, including “Flock”, “Non-flock”, “FlockandNonflock”.

**Species description:** The species of bird studied for each outcome. For some studies which included many species, we describe the location in the original study where species are listed.

**Species level**: whether the study investigated bird abundance and frequency of bird strikes for a single species (“Within species”) or across multiple species (“Between species”)

**Species level coded in analysis:** the code of the species level for each outcome used in the analysis, (“WithinSpecies” / “BetweenSpecies”).

Dependent variable and independent variable information

**Reported bird abundance unit**: describes what unit was used for bird abundance data in our analysis.

**Bird census methods**: methods used to obtain bird abundance data in each study: includes point count, nest count, transect, multiple methods (generally, pulling or estimating abundance data from existing datasets), radar, and mist net.

**Bird census methods coded in analysis:** the code for the degree of bird census methods of each outcome we used in the analysis, including PointCount, NestCount, Transect, MutipleMethods, Radar, and MistNet.

**Reported bird strike unit information**: describes what unit was used for frequency of bird strike data in our analysis.

**Bird strike unit category**: describes whether only bird strikes or total hazardous incidents (only Moreno-Opo & Margalida, 2017) were counted in bird strike frequency data.

**Sampling unit**: The unit compared in original studies. These were primarily temporal (e.g., Month/Year) or across species.

**Degree of sampling unit resolution:** the degree of sampling unit resolution of each outcome, (species/temporal).

**Figures/tables reporting data:** Where abundance and bird strike data for each outcome was collected from the original study.

Variables related to statistics

**Reported sample size**: the sample size for each outcome reported by the original study. “Not reported” means the paper did not report sample size in text.

**Reported r/R^2^**: value reported for correlation statistic in original study.

**Reported P-value**: reported P-value of correlation in original study.

**Reported z**: reported Fisher’s z value in original study.

**Need Recalculation?**: whether the study provides enough information (Pearson’s r/standard error/sample size) for us to pull all data needed for meta-analysis from the original publication (“No”), or whether we needed to extract data to run our own correlation test (“Yes”).

**Need to extract data point from plot?**: if raw data was not provided in articles, we could sometimes extract the data needed from included figures using WebPlotDigitizer. If we needed to extract data from figures, “Yes”, if not, “No”.

**Files saving the extracted dataset(s):** the name of .csv files containing the data we used to analyse each outcome.

**Files saving R code for recalculation:** all code used for our analysis in contained in a single file.

**Recalculated sample size**: sample size for each outcome after we extracted data (if necessary) from the paper and removed data with zero values (see Methods).

**Recalculated r** (Pearson Product moment correlation): the Pearson’s r we calculated for each outcome. Where this value varies from reported values, it is generally the result of our removal of 0 values from abundance data (see Methods).

**Recalculated P-value:** the P-value of the correlation test we got for each outcome following recalculation.

**Fisher's zr**: Fisher’s z of each study, calculated from the Pearson’s r value we generated (or was originally reported).

Variables related to paper quality

**Reported power analysis?**: whether the original study reported an *a priori* power analysis to estimate sample size. No studies in our dataset reported a power analysis.

**Bird abundance data source:** the source of the bird abundance data: Primary (direct measurements); Secondary (measurements reported in other studies, bird surveys, or datasets)

**Bird abundance data source coded in analysis:** the code for the source of bird abundance data of each outcome we used in the analysis, (“Primary” / “Secondary”).

**Strike frequency data source:** the source of the bird strike frequency data: Primary (direct measurements); Secondary (measurements reported in other studies or databases, i.e., FAA Wildlife Strike database).

**Strike frequency data source coded in analysis:** the code for the source of strike frequency data of each outcome we used in the analysis, (“Primary” / “Secondary”).

**Dead birds near runway assumed to have collided with aircraft:** whether dead birds near the runway were assumed to have been struck or not by aircraft in the original study’s strike frequency metric (Yes/Unclear). For studies using strike data pulled from the FAA Wildlife Strike database, this was assumed to be “Yes.” This assumption can over- or under-estimate bird strike frequency data.

**Information on the independence of the sampling units (bird abundance data):** whether the authors provided any details about the independence of the sampling units associated with gathering the bird abundance data. Generally, describes where data comes from or how it was collected.

**Are the bird abundance sampling units independent?:** NA for all, not enough information.

**Information on the independence of the sampling units (strike frequency data):** whether the authors provided any details about the independence of the sampling units associated with gathering the bird abundance data, generally describes where data comes from or how it was collected.

**Are the strike frequency sampling units independent?:** NA for all, not enough information.

**Spatial matching level between bird abundance data and bird strike frequency data:** describes the degree to which bird abundance data matches bird strike frequency data at the spatial level: high match means the same spatial range of data collection for both factors (i.e., both from within the same airport boundary); intermediate match means that there is incomplete overlap in spatial range of data collection for both factors (i.e., abundance data from habitat around airport perimeter, strike data from within airport boundary).

**Spatial matching level between bird abundance data and bird strike frequency data coded in analysis:** the code for spatial matching level between bird abundance data and bird strike frequency data of each outcome we used in the analysis, including (“HighMatch” / “IntermediateMatch”).

**Temporal matching level between bird abundance data and bird strike frequency data**: describes the degree to which bird abundance data matches bird strike frequency data at the temporal level. Two levels: high match, meaning both abundance and strike data were collected during time periods which overlap for >50% of collection period; and low match, meaning both strike and abundance data were collected during time periods which overlap for <50% of collection period.

**Temporal matching level between bird abundance data and bird strike frequency data coded in analysis:** the code for temporal matching level between bird abundance data and bird strike frequency data of each outcome we used in the analysis, (“HighMatch” / “LowMatch”).

**Species matching level between bird abundance data and bird strike frequency data:** describes whether abundance and strike data came from the same species (“high match”). This was the case for all included studies.

**Notes on data selection/manipulation:** These notes describe whether we altered the original dataset in order to include the original study in our meta-analysis.
