# Supplementary material for: Do more birds mean more bird-aircraft collisions? A meta-analysis testing a key wildlife management tenet
Source: PLoS One. 2026 Jul 1;21(7):e0349352. doi: 10.1371/journal.pone.0349352 (PMC13322527; doi:10.1371/journal.pone.0349352)
Supplement: S1 Table — (DOCX) [file pone.0349352.s004.docx]

Table S1: Estimated effect sizes (Fisher’s *z*) and 95% confidence intervals for each level of included moderators. Results are from intercept-removed moderator analyses. Effect sizes for moderator levels whose confidence intervals overlap zero (marked with asterisk) are considered to be no different than zero.

| Moderator Level | Mean Effect Size | Lower Confidence Interval | Upper Confidence Interval |
| --- | --- | --- | --- |
| **Migratory behavior** |  |  |  |
| Non-migratory species only | 0.721 | 0.188 | 1.253 |
| Non-migratory + migratory species | 0.552 | 0.208 | 0.896 |
| **Flocking behavior** |  |  |  |
| Non-flocking species only | 0.823 | 0.096 | 1.550 |
| Flocking species only* | 0.559 | -0.035 | 1.153 |
| Non-flocking + flocking species | 0.594 | 0.234 | 0.953 |
| **Species Level** |  |  |  |
| Within-species | 0.662 | 0.209 | 1.114 |
| Between-species | 0.534 | 0.206 | 0.862 |
| **Degree of spatial resolution** |  |  |  |
| Within airport | 0.583 | 0.252 | 0.915 |
| Between airports | 0.574 | 0.104 | 1.043 |
| **Temporal matching** |  |  |  |
| High match | 0.729 | 0.293 | 1.164 |
| Low/No match | 0.547 | 0.174 | 0.920 |
| **Spatial matching** |  |  |  |
| High match | 0.612 | 0.240 | 0.984 |
| Intermediate match | 0.544 | 0.147 | 0.941 |
| **Bird abundance survey method** |  |  |  |
| Transect | 0.640 | 0.256 | 1.023 |
| Point count | 0.409 | 0.002 | 0.817 |
| Multiple methods* | 0.314 | -0.163 | 0.791 |
| **Bird abundance data source** |  |  |  |
| Primary | 0.680 | 0.356 | 1.004 |
| Secondary* | 0.400 | -0.022 | 0.821 |
| **Bird strike frequency data source** |  |  |  |
| Primary | 0.926 | 0.476 | 1.375 |
| Secondary | 0.429 | 0.145 | 0.713 |
|  |  |  |  |
|  |  |  |  |
|  |  |  |  |
