# Supplementary material for: Do more birds mean more bird-aircraft collisions? A meta-analysis testing a key wildlife management tenet
Source: PLoS One. 2026 Jul 1;21(7):e0349352. doi: 10.1371/journal.pone.0349352 (PMC13322527; doi:10.1371/journal.pone.0349352)
Supplement: Attachment — Submitted filename: Review PONE-D-25-58148.docx [file pone.0349352.s006.docx]

Review MS PONE-D-25-58148

The MS provide a thorough meta-analysis of the world wide relationship between bird abundance and bird strikes and found a positive relationship in the selected case studies. Although widely excepted, this is the first meta-analysis showing the existence of this world wide (studies from all over the world) relationship.

Compliments to the authors, the paper is clear and well-written

Detailed comments

Ln 33-34: An r value of 0.520 is not what you would call very large, probably better than the authors expected.. recommend to remove this sentence from the abstract or rephrase to ‘significant from zero’ or relative terms

Ln 60: See also <https://avisure.com/serious-accident-database/>

Ln 123-127: This search string does not take into account the different papers on bird strike RISK. One might expect that in peer reviewed bird strike risk papers the relationship between bird abundance and strike risk would be the starting point of their model. Very recently, but outside the search scope of this study, a paper was published in Risk Analysis, listing a number of earlier studies of published bird strike risk analysis methods. See <https://doi.org/10.1111/risa.70101>.

Ln 184: Flocking behavior. Please note that flocking behavior is also dependent on bird abundance. For many species hold that with increasing numbers, flock size increases. When numbers are low on an airport, flocking could be limited (and the other way around). Did the authors took this into account?

Ln 234, 239: why is the formula for Z given on a single row and the formula of SE_z_ not?

Ln 453: The different moderators are difficult to read in this table. Highlight the different rows?

Ln 508-511: English language is not the only factor why there’s so little attention to this subject. Both recording most bird strikes (Linnel, 1999: still valid in many places) as well as measuring bird abundance on an airport are lacking. Both are longtime requirements according to ICAO’s Doc9137 and other related documents, but certainly no fulfilled on airports. See further also Ln 536-540 as being helpful for explanations

Ln 629: And to report bird debris for DNA-analysis. If blood smears are under reported a bias in bird strikes will be reported. Like the assumption in the probability of detection of small bird on an airport (your correct statement in Ln 604-610).
