# Supplementary material for: Do more birds mean more bird-aircraft collisions? A meta-analysis testing a key wildlife management tenet
Source: PLoS One. 2026 Jul 1;21(7):e0349352. doi: 10.1371/journal.pone.0349352 (PMC13322527; doi:10.1371/journal.pone.0349352)
Supplement: Attachment — Submitted filename: PLOS.ONE_Reviewer.Response.01.21.26.docx [file pone.0349352.s008.docx]

**Reviewer #1:**

I would like to thank the authors for submitting this interesting and relevant manuscript. The study investigates the relationship between bird abundance and bird strike frequency at airports, and provides valuable insights for wildlife hazard management and aviation safety. Establishing an evidence-based link between local bird abundance and strike occurrence is highly meaningful and may offer an important theoretical foundation for airport bird strike prevention.
However, despite the strengths and relevance of the study, several aspects require clarification, restructuring, or further justification before the manuscript can be considered for publication. My detailed comments are provided below.

**RESPONSE: Thank you very much for the comments, we appreciate the time you have taken to review our manuscript and hope your concerns are fully addressed by the changes we have made and our responses below.**

Major Comments
1. Sample size and heterogeneity
The meta-analysis is based on only 13 studies and 20 effect sizes, with substantial spatial variation and high heterogeneity among studies. This raises concerns about the robustness and representativeness of the conclusions.
Total heterogeneity (I² = 81.07%) and significant Cochran’s Q values across all moderator tests suggest substantial between-study differences.
Many individual effect sizes have small sample sizes (<20), making the reliability of Pearson’s r questionable. For example, Steele (2001) with n = 12 and r = 0.013 (P = 0.968) likely reflects sampling error.
The authors should discuss more explicitly how high heterogeneity and limited sample size may affect the stability and interpretation of the pooled effect.

**RESPONSE: We have added additional discussion of the limited scope of interpretation which should be taken from our pooled effect, given the items you point out here, to the Conclusions section. Also, please, note that Fig. 5 addresses some of the limitations of the current literature when it comes to sample size and associated power. This is relevant to hopefully shift the focus of future work on this topic and increase statistical power of future studies.**

Additionally, the authors may consider whether integrating citizen-science datasets or radar monitoring data could help validate the abundance–strike relationship and compensate for the small number of available studies.

**RESPONSE: This is a fantastic idea, and one that we had considered. However, because meta-analytical methods are primarily used to collate and analyze already published data from which standardized effect sizes can be extracted, it would be inappropriate for us to conduct extra analyses of raw data that did not test directly the association between abundance and bird strike and add those unpublished results to our meta-analysis. So, for us to address this excellent suggestion, we would need to first publish abundance–strike relationship from citizen-science datasets or radar monitoring data in the primary literature. Afterwards, we would be in a position to extract those effect sizes and add them to our meta-analysis.**

2. Geographic imbalance of included studies
The included literature is geographically uneven: seven studies from North America, two each from Australia and Europe, and only one from Africa and Asia. This uneven distribution may influence the generalizability of the results. A discussion on potential geographic bias is needed.

**RESPONSE: We have added caveats related to this geographic bias to the Discussion. This potential bias is unfortunate, but it reflects the literature available.**

3. Presentation of figures and tables
Several issues with figure clarity and table formatting affect readability:
Figures 3, 4, and 5 have low resolution. The colors, labels, and line thickness should be improved to ensure they are legible.

**RESPONSE: Figures have been remade to improve clarity and legibility.**

Figure legends are inserted into the main text while the figures themselves appear at the end. This layout is awkward and disrupts reading flow. The authors should follow standard journal formatting.

**RESPONSE: This formatting follows the guidelines laid out by *PLoS One* here:** [**https://journals.plos.org/plosone/s/submission-guidelines**](https://journals.plos.org/plosone/s/submission-guidelines)

**“Figure captions must be inserted in the text of the manuscript, immediately following the paragraph in which the figure is first cited (read order). Do not include captions as part of the figure files themselves or submit them in a separate document.”**

Figure 4 needs clearer explanations of the meaning of the circle colors and sizes.

**RESPONSE: Circle size reflects the precision (1/SE) of the individual outcome the circle represents. We have added this to Figure 4 legend, and hope that the higher quality/increased legibility of the resubmitted figure makes this clearer. Color is arbitrary: it just visually separates the outcomes from the other moderator levels in the figure.**

Figure 5A appears to offer limited interpretative value, as higher power with larger effect sizes is expected by definition.

**RESPONSE: The utility of Figure 5A, from our perspective, is to collate the powers of all included studies in one place, to better illustrate the overall problem with underpowered studies in this field. Given the reviewer’s concern about the interpretability of effect size values from underpowered studies, and our suggestions that this is a major issue to address moving forward, we believe this is an important figure to retain for the manuscript to make this point to the readers.**

4. Abbreviations
Some abbreviations lack full names at first appearance, such as confidence interval (CI). Please ensure all abbreviations follow standard reporting rules.

**RESPONSE: Thank you for pointing this out. We realized the nonuniform abbreviation of “confidence intervals” throughout the manuscript and chose to remove the CI abbreviation entirely.**

5. Issues with Figure 1
Figure 1 outlines the screening process but does not clearly show the criteria used for study and data selection. Adding explicit inclusion/exclusion criteria would greatly enhance transparency.

**RESPONSE: We have added our inclusion criteria to Figure 1. Please note that these inclusion criteria also appear in the text of the manuscript.**

Table 1 (Moderators): Table 1 structure and logic. Table 1 is confusing and lacks a clear classification framework.
Moderators include biological traits (e.g., migratory behavior), data characteristics (e.g., spatial matching), and methodological factors (e.g., survey method), but these categories are mixed inconsistently.
“Moderator category” uses ecological, confounding factor, and experimental design, but the definitions of these terms are not provided. Readers cannot understand the rationale behind the categorization.
Provide explicit definitions of the moderator categories, including the theoretical or literature basis. Consider reorganizing the table using clearer and more coherent categories such as: Ecological trait moderators, Survey/methodological moderators, Data-matching moderators.
Duplicate moderators and inconsistent entries. Certain moderators (e.g., migratory behavior, flocking behavior, abundance survey method) appear multiple times with inconsistent category assignments. This redundancy makes the table difficult to understand. Each moderator should appear only once, with all corresponding levels listed clearly on the same row.

**RESPONSE: We fully understand the concern, so we have removed Table 1 and instead explain each moderator more thoroughly in the text. Additionally, we agree that moderator categories were ambiguous and did not provide any additional interpretative power; therefore, we have removed them.**

Moderator level definitions unclear. The naming and definitions of moderator levels are inconsistent: Some use binary (high/low), some use three levels (high/intermediate/low), others use primary/secondary, and some use descriptive phrases such as “migratory and non-migratory,” which is ambiguous (is this partial migration?). The criteria for “high match,” “low match,” or “intermediate match” are not defined—are these based on days, weeks, seasons? Spatial distance thresholds? Provide operational definitions for each moderator level. Clarify whether definitions follow previous meta-analyses or are proposed by the authors.

**RESPONSE: The categorical bins for each of the moderators are largely explained in the supplemental appendix (Appendix 2). Where these differ, it is a reflection of our dataset (e.g.,** **for temporal matching, no included studies had an incomplete temporal overlap >50%, rendering an “intermediate” level obsolete).**

**We realized that the migratory behavior moderator was poorly explained and added clarifying text.**

**We have clarified that we established the definitions based on the structure of the dataset we collected. Moreover, we now explicitly define each moderator level in the text and provide clearer rationale for the categorical bin structure(s).**

Standardization of abundance measures. Different studies reported abundance using different metrics: raw counts, model-based density estimates, mixtures of point count and transect data. The manuscript does not explain how these were standardized to ensure comparability. How abundance values were transformed or standardized (raw values, log-transformed, relative abundance, effect-size based)? Whether multi-method sampling introduces bias, and if so, how it was handled.

**RESPONSE: Part of the utility of using a meta-analysis to analyze a system is that they use an effect size, which allows for the calculation of unitless measures of the degree of an effect using raw data (from the primary literature) that is likely to be in different units. We added an explanation in the manuscript about this. This multi-sampling method almost certainly introduces bias into the system, which we discuss at length in the Discussion.**

The table title refers to “type of moderator” while the table column uses “moderator category.”Please unify terminology throughout the manuscript.

**RESPONSE: Table has been removed.**

Table 2. Validity of Pearson correlations with small samples. Several studies have extremely small sample sizes (n < 20), which may make Pearson’s r unreliable or non-representative. This should be discussed.

**RESPONSE: The issue of small sample sizes in several of the included studies is discussed with the results of their respective power. Nevertheless, we have added an explicit clause to our discussion indicating these results in particular should be interpreted with caution.**

**Also note that the effect size used in the meta-analysis was Fisher’s *z*, which normalizes the sampling distribution of a Pearson’s *r* correlation and is generally regarded to be more reliable than Pearson’s *r* for small sample sizes due to the skewness of *r*’s sampling distribution, as you pointed out (see Corey et al. 1998 in *The Journal of General Psychology*, doi:** [**10.1080/00221309809595548**](https://doi.org/10.1080/00221309809595548?urlappend=%3Futm_source%3Dresearchgate.net%26utm_medium%3Darticle)

Clarification of data sources. The table mentions two types of effect-size calculations (a / b). Please clearly annotate which rows correspond to which type.

**RESPONSE: We annotated how Pearson’s r was determined in this table using superscripts associated with each value.**

Missing standard errors. Fisher’s zᵣ values should include corresponding SE values, as these are essential for evaluating precision.

**We have added a column with SE values associated with each Fisher’s z_r_ values.**

Table 3: The table lines are incomplete and should follow standard three-line table format. Despite all moderators being non-significant, the Q-tests for heterogeneity are highly significant. This raises the question: is the lack of significant moderator effects due to genuine homogeneity across levels, or simply insufficient statistical power from small sample sizes and between-study differences? A clearer interpretation is needed.

**RESPONSE: We have removed the statistics (Q, d.f., P-value) for the intercept (original multilevel) model and now report them in the text to remove the unfinished row. We were unsure what the reviewer meant my standard three-line table format, but hope that the formatting changes made (e.g., adding a line to the bottom of the table) adequately address these concerns.**

**Regarding the significant results observed in our Q-tests: our interpretation is that these results are likely driven by both factors you cite in your comment. We know that these studies are underpowered (see Figure 5 in our manuscript and its interpretation), so low sample sizes are certainly contributing to these results. We also observed genuinely high levels of between-study differences (I^2). We have added a line to the Discussion to be clearer in our interpretation: “****It is possible that the apparent homogeneity across moderator levels may result from the low observed power of the included studies (Figure 5) and the high between-study heterogeneity. However, it is not possible to parse out the relative importance of these factors.”**

Minor Comments:
Ensure consistency in italicization and bold formatting for statistical terms and species names.

**RESPONSE: All statistics and species italicization is now standardized.**

Improve the clarity of color schemes and font sizes in all figures.

**RESPONSE: Figures have been remade to improve clarity and legibility.**

Some sentences in the results section are overly long; consider improving readability.

**RESPONSE: We have copyedited the manuscript to remove unnecessarily long sentences in the Results section.**

**Reviewer #2: Review MS PONE-D-25-58148**
The MS provide a thorough meta-analysis of the world wide relationship between bird abundance and bird strikes and found a positive relationship in the selected case studies. Although widely excepted, this is the first meta-analysis showing the existence of this world wide (studies from all over the world) relationship.
Compliments to the authors, the paper is clear and well-written

**RESPONSE: Thank you very much for the compliments, we appreciate the time you have taken to review our manuscript.**

Detailed comments
Ln 33-34: An r value of 0.520 is not what you would call very large, probably better than the authors expected.. recommend to remove this sentence from the abstract or rephrase to ‘significant from zero’ or relative terms

**RESPONSE: We have removed the statement about the relative size of the Pearson’s r value from the Abstract.**

Ln 60: See also <https://avisure.com/serious-accident-database/>

**RESPONSE: Thank you for the resource. We have included the statistics in this database in the Introduction.**

Ln 123-127: This search string does not take into account the different papers on bird strike RISK. One might expect that in peer reviewed bird strike risk papers the relationship between bird abundance and strike risk would be the starting point of their model. Very recently, but outside the search scope of this study, a paper was published in Risk Analysis, listing a number of earlier studies of published bird strike risk analysis methods. See <https://doi.org/10.1111/risa.70101>.

**RESPONSE: We agree that risk is certainly an important factor to consider in the broader sense of prioritizing wildlife management resources and species of concern. For the scope of this paper, studies on risk are not necessarily useful, as they do not report raw abundance data associated with bird strike data for single species in a way that can be broken down and included in our meta-analysis.**

Ln 184: Flocking behavior. Please note that flocking behavior is also dependent on bird abundance. For many species hold that with increasing numbers, flock size increases. When numbers are low on an airport, flocking could be limited (and the other way around). Did the authors took this into account?

**RESPONSE: Thank you for this comment. Together with the comments from Reviewer 1, we realized that our description of how we used flocking behavior as a moderator was not clear and have clarified in the text. To be clear, we were not looking at the size of flocks, but simply whether the species tends to flock or not. The moderator is coded as a binary yes/no, dependent on descriptions of species life history. We did not have enough information from each paper to include flock size.**

Ln 234, 239: why is the formula for Z given on a single row and the formula of SEz not?

**RESPONSE: Thank you for pointing out the formatting inconsistency. We have standardized all formulas to single rows.**

Ln 453: The different moderators are difficult to read in this table. Highlight the different rows?

**RESPONSE: We have added alternating-row coloration to enhance the legibility of this table.**

Ln 508-511: English language is not the only factor why there’s so little attention to this subject. Both recording most bird strikes (Linnel, 1999: still valid in many places) as well as measuring bird abundance on an airport are lacking. Both are longtime requirements according to ICAO’s Doc9137 and other related documents, but certainly no fulfilled on airports. See further also Ln 536-540 as being helpful for explanations

**RESPONSE: Thank you for this suggestion. We have included a mention of reporting compliance, and the biases introduced by non-compliance, into our Discussion, citing Linnel et al. 1999 and the ICAO document.**

Ln 629: And to report bird debris for DNA-analysis. If blood smears are under reported a bias in bird strikes will be reported. Like the assumption in the probability of detection of small bird on an airport (your correct statement in Ln 604-610).

**RESPONSE: This is a good point, especially considering documented biases for other methods (i.e., body size for carcass recovery). We have added this suggestion to the recommendations made in the Discussion.**
