# Supplementary material for: Do more birds mean more bird-aircraft collisions? A meta-analysis testing a key wildlife management tenet
Source: PLoS One. 2026 Jul 1;21(7):e0349352. doi: 10.1371/journal.pone.0349352 (PMC13322527; doi:10.1371/journal.pone.0349352)
Supplement: Attachment — Submitted filename: PLOS One_Response.to.Reviewers_04.17.26.docx [file pone.0349352.s009.docx]

**NOTE ON AN UNPROMTED CHANGE BY THE AUTHORS: In previous versions of this manuscript, we were using the term “meta-regression” to refer to all of our moderator analyses. For moderator analyses of categorical moderators, a more specific term is “subgroup analysis” (see Spineli & Pandis 2020; doi: 10.1016/j.ajodo.2020.09.001). We have changed the language across the manuscript to “subgroup analysis” for analyses investigating a categorical moderator. Notably, subgroup analyses *are* meta-regressions (Nakagawa et al. 2017; doi: 10.1186/s12915-017-0357-7) but we believe this change will help to avoid unnecessary confusion, given the varying connotations of “meta-regression” across the literature.**

Additional Editor Comments:

I would like the authors to consider the following minor comments:

Minor comments

Line 58 – Please include the scientific name of Canada geese (Branta canadensis). Once introduced, the scientific name can be removed from later occurrences (e.g., lines 602 and 668).

**RESPONSE: We have added the scientific name to the first occurrence and removed it from the rest of the manuscript.**

Lines 150–151 – The potential bias associated with including only English-language articles should be more clearly acknowledged as a limitation of the study. Although this issue is briefly mentioned in the Discussion, explicitly addressing it would help clarify that restricting the search to English-language literature may not only reduce the number of studies included in the dataset, but may also lead to an overrepresentation of research conducted in North America. In addition, this approach may overlook relevant research and management experiences published in other languages, which sometimes remain underrepresented in international syntheses due to the predominance of English-language literature.

**RESPONSE: Certainly. In the Discussion section, we have added the points made here to our discussion of the study’s limitations.**

Lines 169–173 – It would be advisable to clearly specify in this paragraph that nine moderators were used in the analyses.

**RESPONSE: Good point, thank you – we now clearly specify “nine moderators” were used in the analyses.**

Following Reviewer 1’s suggestion regarding language clarity, some expressions could be refined. For example, in line 457, the expression “did not have an inordinate effect” could be reviewed for clarity.

**RESPONSE: We hope that our final copy edit has removed all of these clunky and unclear phrases. We have changed the wording of the clause pointed out here to “There was no significant effect”.**

I also note that the Results section contains several extensive explanations in parentheses (e.g., lines 450, 473, 478, 493, 520). While detailed explanations are appreciated, excessive clarification within the Results section may reduce readability. I suggest considering whether some of these explanations could be moved to the Methods section, allowing the Results to remain more concise.

**RESPONSE: We agree that this would improve the flow of the results section. For several of these items, we decided the explanation in parentheses was unnecessary, having already been explained in the Methods; the others have been moved to the Methods section.**

Lines 673–677 – The statement regarding the availability of wildlife strike databases may benefit from some refinement. In Europe, pilots are also required to report bird strikes, and several countries maintain comprehensive databases, often coordinated at the European level. However, these datasets are generally not open access, and researchers typically need to submit an official request to obtain the data. The authors may therefore wish to clarify that the FAA database is one of the most extensive open-access wildlife strike datasets, rather than implying that comparable reporting systems do not exist elsewhere.

**RESPONSE: This is an excellent point. We have clarified the language of this section to reflect this suggestion.**

I look forward to receiving a revised version of your manuscript addressing these points.

**RESPONSE: We would like to sincerely thank the editor for their time in reviewing our manuscript, and the helpful comments.**

Review Comments to the Author

**Reviewer #1:** I would like to thank the authors for their careful and thorough revision of the manuscript. I also congratulate you on conducting, to my knowledge, the first meta-analysis in the field of airport bird strike prevention. This study has clear exploratory value and represents an important step toward evidence-based wildlife hazard management in aviation.

**RESPONSE: We would like to thank the reviewer for their time and pointing out that this is likely the first meta-analysis regarding airport bird strike prevention. We believe this is correct and had not considered this aspect of the novelty of this study.**

In this revised version, the manuscript has been substantially improved. The authors have addressed the major concerns raised in the previous review, particularly regarding heterogeneity, statistical power, moderator clarification, and figure presentation. The structure is clearer, and the discussion now better reflects the limitations of the available literature.

**RESPONSE: Thank you – we are glad we were able to properly address these concerns.**

Please conduct a final careful language check to improve clarity and conciseness in several sections (especially parts of the Results and Discussion), where some sentences remain slightly complex.

**RESPONSE: We have performed a final copy edit to the manuscript, taking care to break up unwieldly and complex sentences.**

I believe this manuscript makes a meaningful contribution to the field and is close to being suitable for publication pending these minor revisions.

**Reviewer #2:** I would like to thank the authors to incorporate the reviewer comments properly into the manuscript.

**RESPONSE: We thank the reviewer for their time in reviewing our manuscript again.**
