# Supplementary material for: Do more birds mean more bird-aircraft collisions? A meta-analysis testing a key wildlife management tenet
Source: PLoS One. 2026 Jul 1;21(7):e0349352. doi: 10.1371/journal.pone.0349352 (PMC13322527; doi:10.1371/journal.pone.0349352)
Supplement: Peer review history

# Peer review: Decision Letter 0

**DOI:** 10.1371/journal.pone.0349352.r001

**Authors:** Juan Manuel Pérez-García

23 Dec 2025

PONE-D-25-58148

Do more birds mean more bird-aircraft collisions? A meta-analysis testing a key wildlife management tenet

PLOS One

Dear Dr. Guenin,

Thank you for submitting your manuscript to PLOS ONE. After careful consideration, we feel that it has merit but does not fully meet PLOS ONE’s publication criteria as it currently stands. Therefore, we invite you to submit a revised version of the manuscript that addresses the points raised during the review process.

It should be noted that particular attention should be paid to the remarks made by Reviewer 1 regarding the sample size, heterogeneity, and spatial bias of the studies included in the meta-analysis.

Please submit your revised manuscript by Feb 06 2026 11:59PM. If you will need more time than this to complete your revisions, please reply to this message or contact the journal office at plosone@plos.org. When you're ready to submit your revision, log on to https://www.editorialmanager.com/pone/ and select the 'Submissions Needing Revision' folder to locate your manuscript file.

Please include the following items when submitting your revised manuscript:

- A letter that responds to each point raised by the academic editor and reviewer(s). You should upload this letter as a separate file labeled 'Response to Reviewers'.
- A marked-up copy of your manuscript that highlights changes made to the original version. You should upload this as a separate file labeled 'Revised Manuscript with Track Changes'.
- An unmarked version of your revised paper without tracked changes. You should upload this as a separate file labeled 'Manuscript'.

If you would like to make changes to your financial disclosure, please include your updated statement in your cover letter. Guidelines for resubmitting your figure files are available below the reviewer comments at the end of this letter.

If applicable, we recommend that you deposit your laboratory protocols in protocols.io to enhance the reproducibility of your results. Protocols.io assigns your protocol its own identifier (DOI) so that it can be cited independently in the future. For instructions see: https://journals.plos.org/plosone/s/submission-guidelines#loc-laboratory-protocols. Additionally, PLOS ONE offers an option for publishing peer-reviewed Lab Protocol articles, which describe protocols hosted on protocols.io. Read more information on sharing protocols at https://plos.org/protocols?utm_medium=editorial-email&utm_source=authorletters&utm_campaign=protocols.

We look forward to receiving your revised manuscript.

Kind regards,

Juan Manuel Pérez-García, PhD

Academic Editor

PLOS One

Journal requirements:

When submitting your revision, we need you to address these additional requirements.

1. Please ensure that your manuscript meets PLOS ONE's style requirements, including those for file naming. The PLOS ONE style templates can be found at

https://journals.plos.org/plosone/s/file?id=wjVg/PLOSOne_formatting_sample_main_body.pdf and

https://journals.plos.org/plosone/s/file?id=ba62/PLOSOne_formatting_sample_title_authors_affiliations.pdf

2. If the reviewer comments include a recommendation to cite specific previously published works, please review and evaluate these publications to determine whether they are relevant and should be cited. There is no requirement to cite these works unless the editor has indicated otherwise.

[Note: HTML markup is below. Please do not edit.]

Reviewers' comments:

Reviewer's Responses to Questions

**Comments to the Author**

1. Is the manuscript technically sound, and do the data support the conclusions?

The manuscript must describe a technically sound piece of scientific research with data that supports the conclusions. Experiments must have been conducted rigorously, with appropriate controls, replication, and sample sizes. The conclusions must be drawn appropriately based on the data presented.

Reviewer #1: Yes

Reviewer #2: Yes

**********

2. Has the statistical analysis been performed appropriately and rigorously?

Reviewer #1: Yes

Reviewer #2: Yes

**********

3. Have the authors made all data underlying the findings in their manuscript fully available?

The PLOS Data policy requires authors to make all data underlying the findings described in their manuscript fully available without restriction, with rare exception (please refer to the Data Availability Statement in the manuscript PDF file). The data should be provided as part of the manuscript or its supporting information, or deposited to a public repository. For example, in addition to summary statistics, the data points behind means, medians and variance measures should be available. If there are restrictions on publicly sharing data—e.g. participant privacy or use of data from a third party—those must be specified.

Reviewer #1: Yes

Reviewer #2: Yes

**********

4. Is the manuscript presented in an intelligible fashion and written in standard English?

PLOS ONE does not copyedit accepted manuscripts, so the language in submitted articles must be clear, correct, and unambiguous. Any typographical or grammatical errors should be corrected at revision, so please note any specific errors here.

Reviewer #1: Yes

Reviewer #2: Yes

**********

5. Review Comments to the Author

Please use the space provided to explain your answers to the questions above. You may also include additional comments for the author, including concerns about dual publication, research ethics, or publication ethics. (Please upload your review as an attachment if it exceeds 20,000 characters)

Reviewer #1: I would like to thank the authors for submitting this interesting and relevant manuscript. The study investigates the relationship between bird abundance and bird strike frequency at airports, and provides valuable insights for wildlife hazard management and aviation safety. Establishing an evidence-based link between local bird abundance and strike occurrence is highly meaningful and may offer an important theoretical foundation for airport bird strike prevention.

However, despite the strengths and relevance of the study, several aspects require clarification, restructuring, or further justification before the manuscript can be considered for publication. My detailed comments are provided below.

Major Comments

1. Sample size and heterogeneity

The meta-analysis is based on only 13 studies and 20 effect sizes, with substantial spatial variation and high heterogeneity among studies. This raises concerns about the robustness and representativeness of the conclusions.

Total heterogeneity (I² = 81.07%) and significant Cochran’s Q values across all moderator tests suggest substantial between-study differences.

Many individual effect sizes have small sample sizes (<20), making the reliability of Pearson’s r questionable. For example, Steele (2001) with n = 12 and r = 0.013 (P = 0.968) likely reflects sampling error.

The authors should discuss more explicitly how high heterogeneity and limited sample size may affect the stability and interpretation of the pooled effect.

Additionally, the authors may consider whether integrating citizen-science datasets or radar monitoring data could help validate the abundance–strike relationship and compensate for the small number of available studies.

2. Geographic imbalance of included studies

The included literature is geographically uneven: seven studies from North America, two each from Australia and Europe, and only one from Africa and Asia. This uneven distribution may influence the generalizability of the results. A discussion on potential geographic bias is needed.

3. Presentation of figures and tables

Several issues with figure clarity and table formatting affect readability:

Figures 3, 4, and 5 have low resolution. The colors, labels, and line thickness should be improved to ensure they are legible.

Figure legends are inserted into the main text while the figures themselves appear at the end. This layout is awkward and disrupts reading flow. The authors should follow standard journal formatting.

Figure 4 needs clearer explanations of the meaning of the circle colors and sizes.

Figure 5A appears to offer limited interpretative value, as higher power with larger effect sizes is expected by definition.

4. Abbreviations

Some abbreviations lack full names at first appearance, such as confidence interval (CI). Please ensure all abbreviations follow standard reporting rules.

5. Issues with Figure 1

Figure 1 outlines the screening process but does not clearly show the criteria used for study and data selection. Adding explicit inclusion/exclusion criteria would greatly enhance transparency.

Table 1 (Moderators): Table 1 structure and logic. Table 1 is confusing and lacks a clear classification framework.

Moderators include biological traits (e.g., migratory behavior), data characteristics (e.g., spatial matching), and methodological factors (e.g., survey method), but these categories are mixed inconsistently.

“Moderator category” uses ecological, confounding factor, and experimental design, but the definitions of these terms are not provided. Readers cannot understand the rationale behind the categorization.

Provide explicit definitions of the moderator categories, including the theoretical or literature basis. Consider reorganizing the table using clearer and more coherent categories such as: Ecological trait moderators, Survey/methodological moderators, Data-matching moderators.

Duplicate moderators and inconsistent entries. Certain moderators (e.g., migratory behavior, flocking behavior, abundance survey method) appear multiple times with inconsistent category assignments. This redundancy makes the table difficult to understand. Each moderator should appear only once, with all corresponding levels listed clearly on the same row.

Moderator level definitions unclear. The naming and definitions of moderator levels are inconsistent: Some use binary (high/low), some use three levels (high/intermediate/low), others use primary/secondary, and some use descriptive phrases such as “migratory and non-migratory,” which is ambiguous (is this partial migration?). The criteria for “high match,” “low match,” or “intermediate match” are not defined—are these based on days, weeks, seasons? Spatial distance thresholds? Provide operational definitions for each moderator level. Clarify whether definitions follow previous meta-analyses or are proposed by the authors.

Standardization of abundance measures. Different studies reported abundance using different metrics: raw counts, model-based density estimates, mixtures of point count and transect data. The manuscript does not explain how these were standardized to ensure comparability. How abundance values were transformed or standardized (raw values, log-transformed, relative abundance, effect-size based)? Whether multi-method sampling introduces bias, and if so, how it was handled.

The table title refers to “type of moderator” while the table column uses “moderator category.”Please unify terminology throughout the manuscript.

Table 2. Validity of Pearson correlations with small samples. Several studies have extremely small sample sizes (n < 20), which may make Pearson’s r unreliable or non-representative. This should be discussed. Clarification of data sources. The table mentions two types of effect-size calculations (a / b). Please clearly annotate which rows correspond to which type. Missing standard errors. Fisher’s zᵣ values should include corresponding SE values, as these are essential for evaluating precision.

Table 3: The table lines are incomplete and should follow standard three-line table format. Despite all moderators being non-significant, the Q-tests for heterogeneity are highly significant. This raises the question: is the lack of significant moderator effects due to genuine homogeneity across levels, or simply insufficient statistical power from small sample sizes and between-study differences? A clearer interpretation is needed.

Minor Comments:

Ensure consistency in italicization and bold formatting for statistical terms and species names.

Improve the clarity of color schemes and font sizes in all figures.

Some sentences in the results section are overly long; consider improving readability.

Reviewer #2: Review MS PONE-D-25-58148

The MS provide a thorough meta-analysis of the world wide relationship between bird abundance and bird strikes and found a positive relationship in the selected case studies. Although widely excepted, this is the first meta-analysis showing the existence of this world wide (studies from all over the world) relationship.

Compliments to the authors, the paper is clear and well-written

Detailed comments

Ln 33-34: An r value of 0.520 is not what you would call very large, probably better than the authors expected.. recommend to remove this sentence from the abstract or rephrase to ‘significant from zero’ or relative terms

Ln 60: See also https://avisure.com/serious-accident-database/

Ln 123-127: This search string does not take into account the different papers on bird strike RISK. One might expect that in peer reviewed bird strike risk papers the relationship between bird abundance and strike risk would be the starting point of their model. Very recently, but outside the search scope of this study, a paper was published in Risk Analysis, listing a number of earlier studies of published bird strike risk analysis methods. See https://doi.org/10.1111/risa.70101.

Ln 184: Flocking behavior. Please note that flocking behavior is also dependent on bird abundance. For many species hold that with increasing numbers, flock size increases. When numbers are low on an airport, flocking could be limited (and the other way around). Did the authors took this into account?

Ln 234, 239: why is the formula for Z given on a single row and the formula of SEz not?

Ln 453: The different moderators are difficult to read in this table. Highlight the different rows?

Ln 508-511: English language is not the only factor why there’s so little attention to this subject. Both recording most bird strikes (Linnel, 1999: still valid in many places) as well as measuring bird abundance on an airport are lacking. Both are longtime requirements according to ICAO’s Doc9137 and other related documents, but certainly no fulfilled on airports. See further also Ln 536-540 as being helpful for explanations

Ln 629: And to report bird debris for DNA-analysis. If blood smears are under reported a bias in bird strikes will be reported. Like the assumption in the probability of detection of small bird on an airport (your correct statement in Ln 604-610).

**********

6. PLOS authors have the option to publish the peer review history of their article (what does this mean?). If published, this will include your full peer review and any attached files.

If you choose “no”, your identity will remain anonymous but your review may still be made public.

**Do you want your identity to be public for this peer review?** For information about this choice, including consent withdrawal, please see our Privacy Policy.

Reviewer #1: No

Reviewer #2: **Yes:**Hans van Gasteren

**********

[NOTE: If reviewer comments were submitted as an attachment file, they will be attached to this email and accessible via the submission site. Please log into your account, locate the manuscript record, and check for the action link "View Attachments". If this link does not appear, there are no attachment files.]

To ensure your figures meet our technical requirements, please review our figure guidelines: https://journals.plos.org/plosone/s/figures

You may also use PLOS’s free figure tool, NAAS, to help you prepare publication quality figures: https://journals.plos.org/plosone/s/figures#loc-tools-for-figure-preparation.

NAAS will assess whether your figures meet our technical requirements by comparing each figure against our figure specifications.

Attachment Submitted filename: Review PONE-D-25-58148.docx

# Author comment: Author response to Decision Letter 1

**DOI:** 10.1371/journal.pone.0349352.r002

24 Jan 2026

We would like to sincerely thank the editor and both reviewers for their time in reviewing our manuscript and the helpful suggestions, which have certainly improved the manuscript. Please note that this response letter is also attached as a .docx file, to optimize legibility.

Reviewer #1:

I would like to thank the authors for submitting this interesting and relevant manuscript. The study investigates the relationship between bird abundance and bird strike frequency at airports, and provides valuable insights for wildlife hazard management and aviation safety. Establishing an evidence-based link between local bird abundance and strike occurrence is highly meaningful and may offer an important theoretical foundation for airport bird strike prevention.

However, despite the strengths and relevance of the study, several aspects require clarification, restructuring, or further justification before the manuscript can be considered for publication. My detailed comments are provided below.

RESPONSE: Thank you very much for the comments, we appreciate the time you have taken to review our manuscript and hope your concerns are fully addressed by the changes we have made and our responses below.

Major Comments

1. Sample size and heterogeneity

The meta-analysis is based on only 13 studies and 20 effect sizes, with substantial spatial variation and high heterogeneity among studies. This raises concerns about the robustness and representativeness of the conclusions.

Total heterogeneity (I² = 81.07%) and significant Cochran’s Q values across all moderator tests suggest substantial between-study differences.

Many individual effect sizes have small sample sizes (<20), making the reliability of Pearson’s r questionable. For example, Steele (2001) with n = 12 and r = 0.013 (P = 0.968) likely reflects sampling error.

The authors should discuss more explicitly how high heterogeneity and limited sample size may affect the stability and interpretation of the pooled effect.

RESPONSE: We have added additional discussion of the limited scope of interpretation which should be taken from our pooled effect, given the items you point out here, to the Conclusions section. Also, please, note that Fig. 5 addresses some of the limitations of the current literature when it comes to sample size and associated power. This is relevant to hopefully shift the focus of future work on this topic and increase statistical power of future studies.

Additionally, the authors may consider whether integrating citizen-science datasets or radar monitoring data could help validate the abundance–strike relationship and compensate for the small number of available studies.

RESPONSE: This is a fantastic idea, and one that we had considered. However, because meta-analytical methods are primarily used to collate and analyze already published data from which standardized effect sizes can be extracted, it would be inappropriate for us to conduct extra analyses of raw data that did not test directly the association between abundance and bird strike and add those unpublished results to our meta-analysis. So, for us to address this excellent suggestion, we would need to first publish abundance–strike relationship from citizen-science datasets or radar monitoring data in the primary literature. Afterwards, we would be in a position to extract those effect sizes and add them to our meta-analysis.

2. Geographic imbalance of included studies

The included literature is geographically uneven: seven studies from North America, two each from Australia and Europe, and only one from Africa and Asia. This uneven distribution may influence the generalizability of the results. A discussion on potential geographic bias is needed.

RESPONSE: We have added caveats related to this geographic bias to the Discussion. This potential bias is unfortunate, but it reflects the literature available.

3. Presentation of figures and tables

Several issues with figure clarity and table formatting affect readability:

Figures 3, 4, and 5 have low resolution. The colors, labels, and line thickness should be improved to ensure they are legible.

RESPONSE: Figures have been remade to improve clarity and legibility.

Figure legends are inserted into the main text while the figures themselves appear at the end. This layout is awkward and disrupts reading flow. The authors should follow standard journal formatting.

RESPONSE: This formatting follows the guidelines laid out by PLoS One here: https://journals.plos.org/plosone/s/submission-guidelines

“Figure captions must be inserted in the text of the manuscript, immediately following the paragraph in which the figure is first cited (read order). Do not include captions as part of the figure files themselves or submit them in a separate document.”

Figure 4 needs clearer explanations of the meaning of the circle colors and sizes.

RESPONSE: Circle size reflects the precision (1/SE) of the individual outcome the circle represents. We have added this to Figure 4 legend, and hope that the higher quality/increased legibility of the resubmitted figure makes this clearer. Color is arbitrary: it just visually separates the outcomes from the other moderator levels in the figure.

Figure 5A appears to offer limited interpretative value, as higher power with larger effect sizes is expected by definition.

RESPONSE: The utility of Figure 5A, from our perspective, is to collate the powers of all included studies in one place, to better illustrate the overall problem with underpowered studies in this field. Given the reviewer’s concern about the interpretability of effect size values from underpowered studies, and our suggestions that this is a major issue to address moving forward, we believe this is an important figure to retain for the manuscript to make this point to the readers.

4. Abbreviations

Some abbreviations lack full names at first appearance, such as confidence interval (CI). Please ensure all abbreviations follow standard reporting rules.

RESPONSE: Thank you for pointing this out. We realized the nonuniform abbreviation of “confidence intervals” throughout the manuscript and chose to remove the CI abbreviation entirely.

5. Issues with Figure 1

Figure 1 outlines the screening process but does not clearly show the criteria used for study and data selection. Adding explicit inclusion/exclusion criteria would greatly enhance transparency.

RESPONSE: We have added our inclusion criteria to Figure 1. Please note that these inclusion criteria also appear in the text of the manuscript.

Table 1 (Moderators): Table 1 structure and logic. Table 1 is confusing and lacks a clear classification framework.

Moderators include biological traits (e.g., migratory behavior), data characteristics (e.g., spatial matching), and methodological factors (e.g., survey method), but these categories are mixed inconsistently.

“Moderator category” uses ecological, confounding factor, and experimental design, but the definitions of these terms are not provided. Readers cannot understand the rationale behind the categorization.

Provide explicit definitions of the moderator categories, including the theoretical or literature basis. Consider reorganizing the table using clearer and more coherent categories such as: Ecological trait moderators, Survey/methodological moderators, Data-matching moderators.

Duplicate moderators and inconsistent entries. Certain moderators (e.g., migratory behavior, flocking behavior, abundance survey method) appear multiple times with inconsistent category assignments. This redundancy makes the table difficult to understand. Each moderator should appear only once, with all corresponding levels listed clearly on the same row.

RESPONSE: We fully understand the concern, so we have removed Table 1 and instead explain each moderator more thoroughly in the text. Additionally, we agree that moderator categories were ambiguous and did not provide any additional interpretative power; therefore, we have removed them.

Moderator level definitions unclear. The naming and definitions of moderator levels are inconsistent: Some use binary (high/low), some use three levels (high/intermediate/low), others use primary/secondary, and some use descriptive phrases such as “migratory and non-migratory,” which is ambiguous (is this partial migration?). The criteria for “high match,” “low match,” or “intermediate match” are not defined—are these based on days, weeks, seasons? Spatial distance thresholds? Provide operational definitions for each moderator level. Clarify whether definitions follow previous meta-analyses or are proposed by the authors.

RESPONSE: The categorical bins for each of the moderators are largely explained in the supplemental appendix (Appendix 2). Where these differ, it is a reflection of our dataset (e.g., for temporal matching, no included studies had an incomplete temporal overlap >50%, rendering an “intermediate” level obsolete).

We realized that the migratory behavior moderator was poorly explained and added clarifying text.

We have clarified that we established the definitions based on the structure of the dataset we collected. Moreover, we now explicitly define each moderator level in the text and provide clearer rationale for the categorical bin structure(s).

Standardization of abundance measures. Different studies reported abundance using different metrics: raw counts, model-based density estimates, mixtures of point count and transect data. The manuscript does not explain how these were standardized to ensure comparability. How abundance values were transformed or standardized (raw values, log-transformed, relative abundance, effect-size based)? Whether multi-method sampling introduces bias, and if so, how it was handled.

RESPONSE: Part of the utility of using a meta-analysis to analyze a system is that they use an effect size, which allows for the calculation of unitless measures of the degree of an effect using raw data (from the primary literature) that is likely to be in different units. We added an explanation in the manuscript about this. This multi-sampling method almost certainly introduces bias into the system, which we discuss at length in the Discussion.

The table title refers to “type of moderator” while the table column uses “moderator category.”Please unify terminology throughout the manuscript.

RESPONSE: Table has been removed.

Table 2. Validity of Pearson correlations with small samples. Several studies have extremely small sample sizes (n < 20), which may make Pearson’s r unreliable or non-representative. This should be discussed.

RESPONSE: The issue of small sample sizes in several of the included studies is discussed with the results of their respective power. Nevertheless, we have added an explicit clause to our discussion indicating these results in particular should be interpreted with caution.

Also note that the effect size used in the meta-analysis was Fisher’s z, which normalizes the sampling distribution of a Pearson’s r correlation and is generally regarded to be more reliable than Pearson’s r for small sample sizes due to the skewness of r’s sampling distribution, as you pointed out (see Corey et al. 1998 in The Journal of General Psychology, doi: 10.1080/00221309809595548

Clarification of data sources. The table mentions two types of effect-size calculations (a / b). Please clearly annotate which rows correspond to which type.

RESPONSE: We annotated how Pearson’s r was determined in this table using superscripts associated with each value.

Missing standard errors. Fisher’s zᵣ values should include corresponding SE values, as these are essential for evaluating precision.

We have added a column with SE values associated with each Fisher’s zr values.

Table 3: The table lines are incomplete and should follow standard three-line table format. Despite all moderators being non-significant, the Q-tests for heterogeneity are highly significant. This raises the question: is the lack of significant moderator effects due to genuine homogeneity across levels, or simply insufficient statistical power from small sample sizes and between-study differences? A clearer interpretation is needed.

RESPONSE: We have removed the statistics (Q, d.f., P-value) for the intercept (original multilevel) model and now report them in the text to remove the unfinished row. We were unsure what the reviewer meant my standard three-line table format, but hope that the formatting changes made (e.g., adding a line to the bottom of the table) adequately address these concerns.

Regarding the significant results observed in our Q-tests: our interpretation is that these results are likely driven by both factors you cite in your comment. We know that these studies are underpowered (see Figure 5 in our manuscript and its interpretation), so low sample sizes are certainly contributing to these results. We also observed genuinely high levels of between-study differences (I^2). We have added a line to the Discussion to be clearer in our interpretation: “It is possible that the apparent homogeneity across moderator levels may result from the low observed power of the included studies (Figure 5) and the high between-study heterogeneity. However, it is not possible to parse out the relative importance of these factors.”

Minor Comments:

Ensure consistency in italicization and bold formatting for statistical terms and species names.

RESPONSE: All statistics and species italicization is now standardized.

Improve the clarity of color schemes and font sizes in all figures.

RESPONSE: Figures have been remade to improve clarity and legibility.

Some sentences in the results section are overly long; consider improving readability.

RESPONSE: We have copyedited the manuscript to remove unnecessarily long sentences in the Results section.

Reviewer #2: Review MS PONE-D-25-58148

The MS provide a thorough meta-analysis of the world wide relationship between bird abundance and bird strikes and found a positive relationship in the selected case studies. Although widely excepted, this is the first meta-analysis showing the existence of this world wide (studies from all over the world) relationship.

Compliments to the authors, the paper is clear and well-written

RESPONSE: Thank you very much for the compliments, we appreciate the time you have taken to review our manuscript.

Detailed comments

Ln 33-34: An r value of 0.520 is not what you would call very large, probably better than the authors expected.. recommend to remove this sentence from the abstract or rephrase to ‘significant from zero’ or relative terms

RESPONSE: We have removed the statement about the relative size of the Pearson’s r value from the Abstract.

Ln 60: See also https://avisure.com/serious-accident-database/

RESPONSE: Thank you for the resource. We have included the statistics in this database in the Introduction.

Ln 123-127: This search string does not take into account the different papers on bird strike RISK. One might expect that in peer reviewed bird strike risk papers the relationship between bird abundance and strike risk would be the starting point of their model. Very recently, but outside the search scope of this study, a paper was published in Risk Analysis, listing a number of earlier studies of published bird strike risk analysis methods. See https://doi.org/10.1111/risa.70101.

RESPONSE: We agree that risk is certainly an important factor to consider in the broader sense of prioritizing wildlife management resources and species of concern. For the scope of this paper, studies on risk are not necessarily useful, as they do not report raw abundance data associated with bird strike data for single species in a way that can be broken down and included in our meta-analysis.

Ln 184: Flocking behavior. Please note that flocking behavior is also dependent on bird abundance. For many species hold that with increasing numbers, flock size increases. When numbers are low on an airport, flocking could be limited (and the other way around). Did the authors took this into account?

RESPONSE: Thank you for this comment. Together with the comments from Reviewer 1, we realized that our description of how we used flocking behavior as a moderator was not cle

Attachment Submitted filename: PLOS.ONE_Reviewer.Response.01.21.26.docx

# Peer review: Decision Letter 1

**DOI:** 10.1371/journal.pone.0349352.r003

**Authors:** Juan Manuel Pérez-García

12 Mar 2026

<div>PONE-D-25-58148R1

Do more birds mean more bird-aircraft collisions? A meta-analysis testing a key wildlife management tenet

PLOS One

Dear Dr. Guenin,

Thank you for submitting your manuscript to PLOS ONE. After careful consideration, we feel that it has merit but does not fully meet PLOS ONE’s publication criteria as it currently stands. Therefore, we invite you to submit a revised version of the manuscript that addresses the points raised during the review process.

Before a final decision can be made, I invite the authors to address the minor comments provided by Reviewer 1, particularly regarding a final language revision to improve clarity and conciseness in parts of the manuscript (especially the Results and Discussion sections), and to consider the additional minor comments provided below

Please submit your revised manuscript by Apr 26 2026 11:59PM. If you will need more time than this to complete your revisions, please reply to this message or contact the journal office at plosone@plos.org. When you're ready to submit your revision, log on to https://www.editorialmanager.com/pone/ and select the 'Submissions Needing Revision' folder to locate your manuscript file.

Please include the following items when submitting your revised manuscript:

- A letter that responds to each point raised by the academic editor and reviewer(s). You should upload this letter as a separate file labeled 'Response to Reviewers'.
- A marked-up copy of your manuscript that highlights changes made to the original version. You should upload this as a separate file labeled 'Revised Manuscript with Track Changes'.
- An unmarked version of your revised paper without tracked changes. You should upload this as a separate file labeled 'Manuscript'.

-->If you would like to make changes to your financial disclosure, please include your updated statement in your cover letter. Guidelines for resubmitting your figure files are available below the reviewer comments at the end of this letter.

If applicable, we recommend that you deposit your laboratory protocols in protocols.io to enhance the reproducibility of your results. Protocols.io assigns your protocol its own identifier (DOI) so that it can be cited independently in the future. For instructions see: https://journals.plos.org/plosone/s/submission-guidelines#loc-laboratory-protocols. Additionally, PLOS ONE offers an option for publishing peer-reviewed Lab Protocol articles, which describe protocols hosted on protocols.io. Read more information on sharing protocols at https://plos.org/protocols?utm_medium=editorial-email&utm_source=authorletters&utm_campaign=protocols.

We look forward to receiving your revised manuscript.

Kind regards,

Juan Manuel Pérez-García, PhD

Academic Editor

PLOS One

Journal Requirements:

If the reviewer comments include a recommendation to cite specific previously published works, please review and evaluate these publications to determine whether they are relevant and should be cited. There is no requirement to cite these works unless the editor has indicated otherwise.

Please review your reference list to ensure that it is complete and correct. If you have cited papers that have been retracted, please include the rationale for doing so in the manuscript text, or remove these references and replace them with relevant current references. Any changes to the reference list should be mentioned in the rebuttal letter that accompanies your revised manuscript. If you need to cite a retracted article, indicate the article’s retracted status in the References list and also include a citation and full reference for the retraction notice.

Additional Editor Comments:

I would like the authors to consider the following minor comments:

Minor comments

Line 58 – Please include the scientific name of Canada geese (Branta canadensis). Once introduced, the scientific name can be removed from later occurrences (e.g., lines 602 and 668).

Lines 150–151 – The potential bias associated with including only English-language articles should be more clearly acknowledged as a limitation of the study. Although this issue is briefly mentioned in the Discussion, explicitly addressing it would help clarify that restricting the search to English-language literature may not only reduce the number of studies included in the dataset, but may also lead to an overrepresentation of research conducted in North America. In addition, this approach may overlook relevant research and management experiences published in other languages, which sometimes remain underrepresented in international syntheses due to the predominance of English-language literature.

Lines 169–173 – It would be advisable to clearly specify in this paragraph that nine moderators were used in the analyses.

Following Reviewer 1’s suggestion regarding language clarity, some expressions could be refined. For example, in line 457, the expression “did not have an inordinate effect” could be reviewed for clarity.

I also note that the Results section contains several extensive explanations in parentheses (e.g., lines 450, 473, 478, 493, 520). While detailed explanations are appreciated, excessive clarification within the Results section may reduce readability. I suggest considering whether some of these explanations could be moved to the Methods section, allowing the Results to remain more concise.

Lines 673–677 – The statement regarding the availability of wildlife strike databases may benefit from some refinement. In Europe, pilots are also required to report bird strikes, and several countries maintain comprehensive databases, often coordinated at the European level. However, these datasets are generally not open access, and researchers typically need to submit an official request to obtain the data. The authors may therefore wish to clarify that the FAA database is one of the most extensive open-access wildlife strike datasets, rather than implying that comparable reporting systems do not exist elsewhere.

I look forward to receiving a revised version of your manuscript addressing these points.

[Note: HTML markup is below. Please do not edit.]

Reviewers' comments:

Reviewer's Responses to Questions

**Comments to the Author**

1. If the authors have adequately addressed your comments raised in a previous round of review and you feel that this manuscript is now acceptable for publication, you may indicate that here to bypass the “Comments to the Author” section, enter your conflict of interest statement in the “Confidential to Editor” section, and submit your "Accept" recommendation.

Reviewer #1: All comments have been addressed

Reviewer #2: All comments have been addressed

**********

2. Is the manuscript technically sound, and do the data support the conclusions?

The manuscript must describe a technically sound piece of scientific research with data that supports the conclusions. Experiments must have been conducted rigorously, with appropriate controls, replication, and sample sizes. The conclusions must be drawn appropriately based on the data presented.

Reviewer #1: Yes

Reviewer #2: Yes

**********

3. Has the statistical analysis been performed appropriately and rigorously?

Reviewer #1: Yes

Reviewer #2: Yes

**********

4. Have the authors made all data underlying the findings in their manuscript fully available?

The PLOS Data policy requires authors to make all data underlying the findings described in their manuscript fully available without restriction, with rare exception (please refer to the Data Availability Statement in the manuscript PDF file). The data should be provided as part of the manuscript or its supporting information, or deposited to a public repository. For example, in addition to summary statistics, the data points behind means, medians and variance measures should be available. If there are restrictions on publicly sharing data—e.g. participant privacy or use of data from a third party—those must be specified.

Reviewer #1: Yes

Reviewer #2: Yes

**********

5. Is the manuscript presented in an intelligible fashion and written in standard English?

PLOS ONE does not copyedit accepted manuscripts, so the language in submitted articles must be clear, correct, and unambiguous. Any typographical or grammatical errors should be corrected at revision, so please note any specific errors here.

Reviewer #1: Yes

Reviewer #2: Yes

**********

6. Review Comments to the Author

Please use the space provided to explain your answers to the questions above. You may also include additional comments for the author, including concerns about dual publication, research ethics, or publication ethics. (Please upload your review as an attachment if it exceeds 20,000 characters)

Reviewer #1: I would like to thank the authors for their careful and thorough revision of the manuscript. I also congratulate you on conducting, to my knowledge, the first meta-analysis in the field of airport bird strike prevention. This study has clear exploratory value and represents an important step toward evidence-based wildlife hazard management in aviation.

In this revised version, the manuscript has been substantially improved. The authors have addressed the major concerns raised in the previous review, particularly regarding heterogeneity, statistical power, moderator clarification, and figure presentation. The structure is clearer, and the discussion now better reflects the limitations of the available literature.

Please conduct a final careful language check to improve clarity and conciseness in several sections (especially parts of the Results and Discussion), where some sentences remain slightly complex.

I believe this manuscript makes a meaningful contribution to the field and is close to being suitable for publication pending these minor revisions.

Reviewer #2: I would like to thank the authors to incorporate the reviewer comments properly into the manuscript.

**********

7. PLOS authors have the option to publish the peer review history of their article (what does this mean?). If published, this will include your full peer review and any attached files.

If you choose “no”, your identity will remain anonymous but your review may still be made public.

**Do you want your identity to be public for this peer review?** For information about this choice, including consent withdrawal, please see our Privacy Policy.

Reviewer #1: No

Reviewer #2: No

**********

[NOTE: If reviewer comments were submitted as an attachment file, they will be attached to this email and accessible via the submission site. Please log into your account, locate the manuscript record, and check for the action link "View Attachments". If this link does not appear, there are no attachment files.]

To ensure your figures meet our technical requirements, please review our figure guidelines: https://journals.plos.org/plosone/s/figures

You may also use PLOS’s free figure tool, NAAS, to help you prepare publication quality figures: https://journals.plos.org/plosone/s/figures#loc-tools-for-figure-preparation.

NAAS will assess whether your figures meet our technical requirements by comparing each figure against our figure specifications.

# Author comment: Author response to Decision Letter 2

**DOI:** 10.1371/journal.pone.0349352.r004

17 Apr 2026

NOTE ON AN UNPROMTED CHANGE BY THE AUTHORS: In previous versions of this manuscript, we were using the term “meta-regression” to refer to all of our moderator analyses. For moderator analyses of categorical moderators, a more specific term is “subgroup analysis” (see Spineli & Pandis 2020; doi: 10.1016/j.ajodo.2020.09.001). We have changed the language across the manuscript to “subgroup analysis” for analyses investigating a categorical moderator. Notably, subgroup analyses are a type of meta-regression, (Nakagawa et al. 2017; doi: 10.1186/s12915-017-0357-7) but we believe this change will help to avoid unnecessary confusion, given the varying connotations of “meta-regression” across the literature.

Additional Editor Comments:

I would like the authors to consider the following minor comments:

Minor comments

Line 58 – Please include the scientific name of Canada geese (Branta canadensis). Once introduced, the scientific name can be removed from later occurrences (e.g., lines 602 and 668).

RESPONSE: We have added the scientific name to the first occurrence and removed it from the rest of the manuscript.

Lines 150–151 – The potential bias associated with including only English-language articles should be more clearly acknowledged as a limitation of the study. Although this issue is briefly mentioned in the Discussion, explicitly addressing it would help clarify that restricting the search to English-language literature may not only reduce the number of studies included in the dataset, but may also lead to an overrepresentation of research conducted in North America. In addition, this approach may overlook relevant research and management experiences published in other languages, which sometimes remain underrepresented in international syntheses due to the predominance of English-language literature.

RESPONSE: Certainly. In the Discussion section, we have added the points made here to our discussion of the study’s limitations.

Lines 169–173 – It would be advisable to clearly specify in this paragraph that nine moderators were used in the analyses.

RESPONSE: Good point, thank you – we now clearly specify “nine moderators” were used in the analyses.

Following Reviewer 1’s suggestion regarding language clarity, some expressions could be refined. For example, in line 457, the expression “did not have an inordinate effect” could be reviewed for clarity.

RESPONSE: We hope that our final copy edit has removed all of these clunky and unclear phrases. We have changed the wording of the clause pointed out here to “There was no significant effect”.

I also note that the Results section contains several extensive explanations in parentheses (e.g., lines 450, 473, 478, 493, 520). While detailed explanations are appreciated, excessive clarification within the Results section may reduce readability. I suggest considering whether some of these explanations could be moved to the Methods section, allowing the Results to remain more concise.

RESPONSE: We agree that this would improve the flow of the results section. For several of these items, we decided the explanation in parentheses was unnecessary, having already been explained in the Methods; the others have been moved to the Methods section.

Lines 673–677 – The statement regarding the availability of wildlife strike databases may benefit from some refinement. In Europe, pilots are also required to report bird strikes, and several countries maintain comprehensive databases, often coordinated at the European level. However, these datasets are generally not open access, and researchers typically need to submit an official request to obtain the data. The authors may therefore wish to clarify that the FAA database is one of the most extensive open-access wildlife strike datasets, rather than implying that comparable reporting systems do not exist elsewhere.

RESPONSE: This is an excellent point. We have clarified the language of this section to reflect this suggestion.

I look forward to receiving a revised version of your manuscript addressing these points.

RESPONSE: We would like to sincerely thank the editor for their time in reviewing our manuscript, and the helpful comments.

Review Comments to the Author

Reviewer #1: I would like to thank the authors for their careful and thorough revision of the manuscript. I also congratulate you on conducting, to my knowledge, the first meta-analysis in the field of airport bird strike prevention. This study has clear exploratory value and represents an important step toward evidence-based wildlife hazard management in aviation.

RESPONSE: We would like to thank the reviewer for their time and pointing out that this is likely the first meta-analysis regarding airport bird strike prevention. We believe this is correct and had not considered this aspect of the novelty of this study.

In this revised version, the manuscript has been substantially improved. The authors have addressed the major concerns raised in the previous review, particularly regarding heterogeneity, statistical power, moderator clarification, and figure presentation. The structure is clearer, and the discussion now better reflects the limitations of the available literature.

RESPONSE: Thank you – we are glad we were able to properly address these concerns.

Please conduct a final careful language check to improve clarity and conciseness in several sections (especially parts of the Results and Discussion), where some sentences remain slightly complex.

RESPONSE: We have performed a final copy edit to the manuscript, taking care to break up unwieldly and complex sentences.

I believe this manuscript makes a meaningful contribution to the field and is close to being suitable for publication pending these minor revisions.

Reviewer #2: I would like to thank the authors to incorporate the reviewer comments properly into the manuscript.

RESPONSE: We thank the reviewer for their time in reviewing our manuscript again.

Attachment Submitted filename: PLOS One_Response.to.Reviewers_04.17.26.docx

# Editor report: Decision Letter 2

**DOI:** 10.1371/journal.pone.0349352.r005

**Authors:** Juan Manuel Pérez-García

29 Apr 2026

Do more birds mean more bird-aircraft collisions? A meta-analysis testing a key wildlife management tenet

PONE-D-25-58148R2

Dear Dr. Guenin,

We’re pleased to inform you that your manuscript has been judged scientifically suitable for publication and will be formally accepted for publication once it meets all outstanding technical requirements.

Within one week, you’ll receive an e-mail detailing the required amendments. When these have been addressed, you’ll receive a formal acceptance letter and your manuscript will be scheduled for publication.

An invoice will be generated when your article is formally accepted. Please note, if your institution has a publishing partnership with PLOS and your article meets the relevant criteria, all or part of your publication costs will be covered. Please make sure your user information is up-to-date by logging into Editorial Manager at Editorial Manager® and clicking the ‘Update My Information' link at the top of the page. For questions related to billing, please contact billing support.

If your institution or institutions have a press office, please notify them about your upcoming paper to help maximize its impact. If they’ll be preparing press materials, please inform our press team as soon as possible -- no later than 48 hours after receiving the formal acceptance. Your manuscript will remain under strict press embargo until 2 pm Eastern Time on the date of publication. For more information, please contact onepress@plos.org.

Kind regards,

Juan Manuel Pérez-García, PhD

Academic Editor

PLOS One

Additional Editor Comments (optional):

Reviewers' comments:

# Editor report: Acceptance letter

**DOI:** 10.1371/journal.pone.0349352.r006

**Authors:** Juan Manuel Pérez-García

PONE-D-25-58148R2

PLOS One

Dear Dr. Guenin,

I'm pleased to inform you that your manuscript has been deemed suitable for publication in PLOS One. Congratulations! Your manuscript is now being handed over to our production team.

At this stage, our production department will prepare your paper for publication. This includes ensuring the following:

* All references, tables, and figures are properly cited

* All relevant supporting information is included in the manuscript submission,

* There are no issues that prevent the paper from being properly typeset

You will receive further instructions from the production team, including instructions on how to review your proof when it is ready. Please keep in mind that we are working through a large volume of accepted articles, so please give us a few days to review your paper and let you know the next and final steps.

Lastly, if your institution or institutions have a press office, please let them know about your upcoming paper now to help maximize its impact. If they'll be preparing press materials, please inform our press team within the next 48 hours. Your manuscript will remain under strict press embargo until 2 pm Eastern Time on the date of publication. For more information, please contact onepress@plos.org.

You will receive an invoice from PLOS for your publication fee after your manuscript has reached the completed accept phase. If you receive an email requesting payment before acceptance or for any other service, this may be a phishing scheme. Learn how to identify phishing emails and protect your accounts at https://explore.plos.org/phishing.

If we can help with anything else, please email us at customercare@plos.org.

Thank you for submitting your work to PLOS ONE and supporting open access.

Kind regards,

PLOS ONE Editorial Office Staff

on behalf of

Dr. Juan Manuel Pérez-García

Academic Editor

PLOS One
